# Topical Hyaluronic Acid for Palatal Donor-Site Healing After Autogenous Gingival Graft Harvesting: A Systematic Review and Time-Specific Meta-Analysis with Clinical Implications for Postoperative Care

**DOI:** 10.3390/jpm16100492

**Published:** 2026-09-23

**Authors:** Luis Chauca-Bajaña, Hector Alfredo Lema Gutierrez, Gabriela Guadalupe Zambrano-Manzaba, Nayely Jimena Teran-Sanchez, Andrea Ordoñez-Balladares, Rosangela Caicedo-Quiroz, Roberto Tolozano-Benites, Marcela Alejandra Ordoñez Balladares, Carlos Carpio Cevallos, Byron Velasquez Ron

**Affiliations:** 1 College Dentistry, University Bolivariana del Ecuador, Durán 092406, Ecuador; luis.chaucab@ug.edu.ec (L.C.-B.); rcaicedoq@ube.edu.ec (R.C.-Q.); rtolozano@ube.edu.ec (R.T.-B.);; 2 College of Dentistry, University of Guayaquil, Guayaquil 090101, Ecuador; 3 Faculty of Health Sciences, Universidad Católica de Santiago de Guayaquil (UCSG), Guayas 090101, Ecuador; 4 School of Dentistry, Universidad Católica de Santiago de Guayaquil (UCSG), Guayas 090101, Ecuador; 5 Cátedra Hispanoamericana de Investigación en Implantología Clínica y Prótesis Dental, Facultad de Ciencias de la Salud, Universidad Católica de Santiago de Guayaquil (UCSG)–SOADCO, Guayaquil 090615, Ecuador; 6 Grupo de Investigación de Prevención y Salud Oral, Universidad Católica de Santiago de Guayaquil (UCSG), Guayas 090101, Ecuador; 7 Carrera de Odontología, Universidad de Las Américas (UDLA) Ecuador, Quito 170102, Ecuador; byron.velasquez@udla.edu.ec

**Keywords:** hyaluronic acid, palatal wound healing, gingival graft, postoperative pain, systematic review

## Abstract

**Background**: Palatal graft harvesting can cause pain, bleeding, and delayed healing, with postoperative recovery varying across patients and over time. Topical hyaluronic acid (HA) may reduce donor-site morbidity and promote tissue repair. Identifying the postoperative time points and clinical domains in which HA may provide benefit could support more informed, patient-centered postoperative management. **Objective**: To evaluate the time-specific effects of topical HA on palatal wound healing and postoperative morbidity after autogenous gingival graft harvesting and to examine their clinical implications for postoperative care. **Methods**: Randomized clinical trials comparing topical HA with placebo, no application, or standard care were reviewed. Separate random-effects meta-analyses were performed at each postoperative time point using risk ratios (RRs) for dichotomous outcomes and mean differences (MDs) for continuous outcomes. Between-study variance was estimated using REML. Leave-one-out, Hartung–Knapp, and formulation-based sensitivity analyses were performed. Risk of bias was assessed using RoB 2, and certainty of evidence using GRADE. **Results**: Eight randomized trials were included. HA increased complete epithelialization at day 14 (RR = 2.11; 95% CI, 1.13–3.94) and day 21 (RR = 1.62; 95% CI, 1.29–2.03). WHI favored HA at day 7 (MD = 0.58; 95% CI, 0.42–0.75). Pain reductions at days 3 and 7 and bleeding reductions at days 1 and 3 were not statistically significant. Analgesic consumption was lower at day 3 (MD = −0.80; 95% CI, −1.34 to −0.26), while color match favored HA at days 14 and 21. Hartung–Knapp analyses increased uncertainty for WHI, pain, and color match. No result was rated at high overall risk of bias; certainty ranged from low to very low. **Conclusions**: Topical HA appears to provide its most consistent benefit in accelerating palatal epithelialization and selected healing outcomes rather than uniformly reducing all dimensions of postoperative morbidity. These time- and outcome-specific effects may inform patient-centered and risk-adapted postoperative strategies, particularly when early wound closure is a clinical priority. However, the current aggregate evidence does not permit identification of individual treatment responders. Future adequately powered randomized trials incorporating standardized HA formulations, patient-level characteristics, potential effect modifiers, and harmonized outcome assessment are needed to support precision treatment selection.

## 1. Introduction

Autogenous soft-tissue grafting remains a reference treatment in periodontal and peri-implant plastic surgery because of its predictable ability to increase gingival thickness, augment keratinized tissue, cover exposed roots, and reconstruct peri-implant soft-tissue deficiencies [1]. The hard palate is the most frequently used donor site because it provides tissue with favorable biological and structural characteristics. However, graft harvesting creates a second surgical wound and may result in clinically relevant donor-site morbidity [2,3]. This is particularly evident after free gingival graft harvesting, in which the open palatal wound heals predominantly by secondary intention. Postoperative pain, burning sensation, bleeding, delayed epithelialization, eating difficulties, and functional discomfort may negatively affect patient acceptance and overall treatment experience [2,3,4]. Palatal wound healing is a dynamic process involving overlapping hemostatic, inflammatory, proliferative, epithelialization, and remodeling phases [2]. Considerable variability also exists in the outcomes and instruments used to evaluate recovery, including pain intensity, analgesic consumption, wound dimensions, complete epithelialization, clinical healing indices, tissue color match, bleeding, and patient-reported morbidity [4].

Hyaluronic acid, also known as hyaluronan or sodium hyaluronate, is a naturally occurring, non-sulfated glycosaminoglycan and an essential component of the extracellular matrix. Its hygroscopic and viscoelastic properties contribute to tissue hydration, structural stability, and the maintenance of an extracellular environment favorable to cellular migration [5,6,7]. During tissue repair, hyaluronic acid participates in the regulation of inflammation, granulation-tissue organization, angiogenesis, epithelial migration, fibroblast proliferation, collagen deposition, and extracellular-matrix remodeling [5,6,7]. Its biological effects are influenced by molecular weight, concentration, degradation, cross-linking, and interactions with cellular receptors [6,7]. High-molecular-weight hyaluronic acid is generally associated with tissue homeostasis and modulation of inflammation, whereas lower-molecular-weight fragments may function as signaling molecules during the inflammatory and proliferative phases of wound healing [6,7]. In human palatal and gingival fibroblasts, hyaluronic acid formulations have been shown to maintain cell viability, enhance proliferation and migration, and increase the expression of genes associated with type III collagen production and wound healing [8]. These characteristics, together with its biocompatibility and ease of topical application, provide a biological rationale for its use in the management of oral surgical wounds [9].

The present review deliberately focused on HA-based interventions because HA represents a biologically distinct therapeutic approach with a growing body of randomized clinical evidence in palatal donor-site healing. Combining HA with unrelated interventions, such as platelet concentrates, anti-inflammatory drugs, antiseptic agents, or other wound dressings, would address a broader comparative-effectiveness question and would prevent the specific contribution of HA from being estimated reliably. Therefore, the primary quantitative syntheses included interventions in which HA was the sole active therapeutic component, irrespective of the presence of an inert carrier or delivery excipient. Formulations containing another potentially active ingredient whose independent effect could not be isolated were considered only in sensitivity or narrative analyses. This focused approach was intended to improve clinical interpretability and preserve attribution of the pooled effects specifically to topical HA.

Several randomized clinical trials have evaluated topical hyaluronic acid after palatal soft-tissue graft harvesting. An early randomized trial reported lower postoperative pain and burning sensation and earlier complete epithelialization after the application of 0.2% and 0.8% hyaluronic acid gels compared with periodontal dressing alone [10]. Subsequent studies investigated its effects on postoperative pain, analgesic consumption, wound dimensions, clinical healing, epithelialization, and tissue color match [11,12,13]. However, the findings have not been entirely consistent. Some trials reported reductions in pain and analgesic consumption or improvements in epithelialization and clinical healing [10,13], whereas a quasi-randomized trial evaluating a formulation containing green tea extract and hyaluronic acid did not demonstrate additional benefits for pain or wound healing [14]. More recent randomized trials evaluating polyvinylpyrrolidone–sodium hyaluronate gels and standardized carrier systems have reported potentially favorable effects on pain, analgesic consumption, complete epithelialization, clinical healing indices, bleeding, patient satisfaction, and oral health-related quality of life [15,16,17]. A recent randomized crossover trial using standardized palatal wounds has further expanded the evidence by providing repeated patient-reported and clinical measurements throughout the healing period [18]. Nevertheless, differences in hyaluronic acid concentration, molecular characteristics, vehicles, application frequency, cointerventions, control groups, outcome definitions, and follow-up schedules complicate the interpretation of individual studies.

A 2024 systematic review specifically addressing hyaluronic acid for palatal donor-site healing identified eight eligible studies and reported generally favorable results; however, the available evidence was synthesized narratively, without a pooled quantitative effect estimate [19]. A broader network meta-analysis compared several agents used for palatal wound management, but it was not designed to isolate the time-dependent effects of hyaluronic acid across repeated postoperative assessments [20]. Moreover, additional randomized trials have subsequently expanded the evidence base [15,16,17,18]. Therefore, an updated quantitative synthesis is needed to determine the magnitude and temporal pattern of the effects of topical hyaluronic acid while accounting for multiple dependent observations reported within the same study. Accordingly, this systematic review and time-specific meta-analysis aimed to determine whether topical hyaluronic acid reduces postoperative morbidity and improves clinical healing of palatal donor sites after autogenous gingival graft harvesting. A secondary objective was to explore whether the magnitude of the treatment effect differed across postoperative time points, hyaluronic acid formulations, delivery systems, application protocols, graft-harvesting methods, and control interventions.

## 2. Materials and Methods

### 2.1. Protocol and Registrations1

A predefined protocol was developed for the literature search, study selection, data extraction, risk-of-bias assessment, and statistical analysis procedures. The review was designed and reported in accordance with the Preferred Reporting Items for Systematic Reviews and Meta-Analyses (PRISMA 2020) guidelines [21] (Figure 1). The protocol was prospectively registered in the International Prospective Register of Systematic Reviews (PROSPERO) under registration number CRD420261485015 before the formal study-selection and data-extraction procedures. The PRISMA flow diagram describing the study-selection process is presented in Figure 1, and the completed PRISMA 2020 checklist is provided in the Supplementary Materials (Table S1). The final electronic database search was conducted in April 2026.

### 2.2. Focused Question

In adults undergoing autogenous gingival graft harvesting from the palate, does the topical application of hyaluronic acid, compared with placebo, no application, vehicle alone, or standard donor-site care, reduce postoperative morbidity and improve palatal wound healing?

P (Population): Adults undergoing harvesting of an autogenous gingival or soft-tissue graft from the palatal donor site for periodontal or peri-implant plastic surgical procedures.

I (Intervention): Topical application of hyaluronic acid, hyaluronan, or sodium hyaluronate, administered as a gel, mucoadhesive preparation, impregnated sponge, dressing, or another locally delivered formulation.

C (Comparison): Placebo, no topical intervention, spontaneous healing, vehicle or carrier without hyaluronic acid, or standard palatal donor-site care. Active comparators were considered separately when they allowed the specific effect of hyaluronic acid to be evaluated.

O (Outcomes): The primary outcomes were postoperative pain and complete wound epithelialization. Secondary outcomes included clinical wound-healing scores, reduction in wound area, analgesic consumption, burning sensation, tissue color match, postoperative bleeding, patient satisfaction, oral health-related quality of life, and adverse events.

### 2.3. Information Sources and Search Strategy

A comprehensive and structured literature search was conducted in accordance with the PRISMA 2020 recommendations. The following electronic databases and information sources were searched: MEDLINE via PubMed, Embase via Ovid, Web of Science Core Collection, Scopus, the Cochrane Central Register of Controlled Trials (CENTRAL), ClinicalTrials.gov, and the World Health Organization Global Index Medicus regional databases, including African Index Medicus (AIM), Latin American and Caribbean Health Sciences Literature (LILACS), Index Medicus for the Eastern Mediterranean Region (IMEMR), Index Medicus for the South-East Asia Region (IMSEAR), and Western Pacific Region Index Medicus (WPRIM). The Conference Proceedings Citation Index was also searched to identify conference abstracts, unpublished data, and potentially eligible studies not indexed in the principal databases.

Controlled vocabulary terms, including Medical Subject Headings (MeSH) in MEDLINE and Emtree terms in Embase, were combined with free-text keywords using Boolean operators. The principal concepts included hyaluronic acid and palatal donor-site healing after autogenous gingival graft harvesting. The main controlled terms included “Hyaluronic Acid,” “Palate,” “Gingiva/transplantation,” “Connective Tissue/transplantation,” “Transplant Donor Site,” “Wound Healing,” and “Postoperative Pain.” Additional free-text terms included hyaluronan, sodium hyaluronate, cross-linked hyaluronic acid, hyaluronic acid gel, palatal wound, palatal donor site, free gingival graft, connective tissue graft, soft-tissue graft, graft harvesting, wound epithelialization, postoperative pain, patient morbidity, and wound-healing index.

The core PubMed search strategy was structured as follows:

(“Hyaluronic Acid”[MeSH Terms] OR “hyaluronic acid”[Title/Abstract] OR hyaluronan[Title/Abstract] OR “sodium hyaluronate”[Title/Abstract]) AND (“Palate”[MeSH Terms] OR palatal[Title/Abstract] OR palate[Title/Abstract]) AND (“Gingival Transplantation”[MeSH Terms] OR “Transplant Donor Site”[MeSH Terms] OR “free gingival graft”[Title/Abstract] OR “connective tissue graft”[Title/Abstract] OR “soft tissue graft”[Title/Abstract] OR “donor site”[Title/Abstract] OR harvesting[Title/Abstract] OR wound[Title/Abstract]).

The search syntax was adapted to the indexing rules and interface requirements of each database. No restrictions were imposed regarding publication year or language. No methodological filter for randomized trials was applied during the initial search to maximize sensitivity and reduce the risk of excluding potentially eligible studies. The final electronic database search was conducted in April 2026. The complete search strategies for all databases, including the final search date and the number of records retrieved from each source, are provided in Supplementary Table S2.

All identified records were exported to a reference-management program for duplicate removal and subsequently uploaded to the Rayyan QCRI platform (Qatar Computing Research Institute, Doha, Qatar) for title-and-abstract screening and full-text eligibility assessment. To ensure completeness, the reference lists of all included articles and relevant narrative, scoping, and systematic reviews were manually examined. Forward citation tracking of the included studies was performed using Scopus and Web of Science. Clinical trial registries were also examined to identify ongoing, completed but unpublished, or retrospectively registered trials. This process was complemented by a targeted manual search of peer-reviewed journals specializing in periodontology, implant dentistry, oral surgery, and wound healing.

### 2.4. Eligibility Criteria

Studies were selected according to the Population, Intervention, Comparison, Outcomes, and Study design framework.

#### 2.4.1. Inclusion Criteria

1. Study design: Parallel-group, split-mouth, or crossover randomized controlled clinical trials. Multi-arm trials were eligible when they included at least one relevant comparison between a hyaluronic acid intervention and an appropriate control group.
2. Population: Adults aged ≥18 years undergoing harvesting of an autogenous gingival or soft-tissue graft from the hard palate for periodontal or peri-implant plastic surgical procedures. Studies using a standardized palatal wound specifically designed to reproduce free gingival graft harvesting were also considered eligible but were evaluated separately in sensitivity analyses.
3. Intervention: Topical or locally delivered hyaluronic acid, hyaluronan, or sodium hyaluronate applied to the palatal donor site as a gel, mucoadhesive preparation, impregnated sponge, wound dressing, or another local-delivery formulation. Hyaluronic acid could be used alone or over an identical background intervention received by both study groups.
4. Comparison: Placebo, no topical application, spontaneous healing, vehicle or carrier without hyaluronic acid, or standard donor-site care. Comparisons against another active intervention were eligible only when the study also included an appropriate control group or provided data allowing the specific effect of hyaluronic acid to be estimated.
5. Outcomes: Studies were required to report at least one quantitative clinical or patient-reported outcome related to palatal donor-site healing or postoperative morbidity. Eligible outcomes included postoperative pain, complete epithelialization, clinical wound-healing scores, wound area or wound-size reduction, analgesic consumption, burning sensation, tissue color match, postoperative bleeding, patient satisfaction, oral health-related quality of life, or adverse events.
6. Data reporting: Studies had to provide sufficient quantitative information to calculate an effect estimate, such as means and standard deviations, medians and dispersion measures that could be appropriately converted, numbers of participants experiencing an event, change scores, or other reconstructable numerical data.
7. Publication characteristics: Full-text, peer-reviewed original research articles were eligible. No restrictions were imposed regarding publication year or language. When necessary, potentially eligible non-English articles were translated for assessment.

#### 2.4.2. Exclusion Criteria

1. Non-randomized clinical studies, observational studies, case reports, case series, laboratory studies, animal studies, in vitro investigations, systematic reviews, meta-analyses, narrative reviews, editorials, letters, protocols, and conference abstracts without sufficient full-text data.
2. Quasi-randomized studies in which treatment allocation was based on alternation, date of attendance, record number, operator decision, or another predictable method. These studies could be retained for a separate sensitivity or narrative analysis but were excluded from the primary meta-analysis.
3. Studies involving pediatric participants or mixed populations when data for adults could not be extracted separately.
4. Studies evaluating hyaluronic acid exclusively at the graft recipient site without its application to the palatal donor wound.
5. Studies investigating hyaluronic acid in non-palatal oral wounds, such as extraction sockets, gingivectomy sites, periodontal intrabony defects, oral ulcers, or implant surgical sites, without separate data for a palatal graft-harvesting wound.
6. Studies using systemic, intra-articular, or injectable hyaluronic acid without topical or local application to the palatal donor site.
7. Studies evaluating multi-component formulations in which hyaluronic acid was combined with another active agent and the independent effect of hyaluronic acid could not be isolated. These studies could be discussed narratively but were excluded from the primary quantitative synthesis.
8. Studies without an appropriate comparator or without sufficient quantitative information to calculate an effect estimate after reasonable attempts to obtain missing data from the study authors.
9. Duplicate or overlapping publications reporting the same participant sample. In such cases, the most complete publication or the report with the longest relevant follow-up was retained, while companion reports were used only to obtain supplementary information.

### 2.5. Study Selection Process and Data Extraction

Two reviewers (L.C.H. and B.V.R.) independently conducted the literature search, study selection, and eligibility assessment. Following duplicate removal, the titles and abstracts of all retrieved records were screened against the predefined eligibility criteria. Articles considered potentially relevant were subsequently evaluated in full text to determine final inclusion. Reasons for exclusion at the full-text stage were documented. Any disagreement was resolved through discussion and consensus; when consensus could not be reached, a third reviewer (A.O.B.), who was not involved in the initial screening process, acted as an adjudicator.

Data were independently extracted using a standardized and pilot-tested form specifically developed for this systematic review. The following information was collected from each study: first author, publication year, country, randomized clinical trial design, masking procedures, number of study arms, randomized and analyzed sample sizes, characteristics of the topical hyaluronic acid (HA) intervention, HA concentration and formulation, molecular-weight information when available, carrier or dressing material, application frequency and duration, comparator intervention, concomitant postoperative care, follow-up schedule, outcome definitions and assessment methods, adverse events, and principal findings.

For quantitative synthesis, arm-level data were extracted separately for each eligible follow-up time. For dichotomous outcomes, including complete epithelialization and postoperative bleeding, the total number of participants and number of events in each group were recorded. For continuous outcomes, including the Wound Healing Index (WHI), postoperative pain, analgesic consumption, and color match, the sample size, mean, and standard deviation were extracted for the HA and control groups. Information reported as medians, interquartile ranges, or minimum–maximum values was recorded but was not incorporated into the primary mean-difference analyses unless an appropriate transformation had been prespecified. Visual analogue scales reported from 0 to 100 were converted to a common 0-to-10 scale by dividing both the mean and standard deviation by 10. Analgesic consumption was pooled using the mean difference only when studies reported the daily number of tablets administered at the same dose.

For multi-arm trials, only the eligible HA and control groups were included in the primary comparisons; unrelated active comparator groups, such as MEBO, injectable platelet-rich fibrin, hypochlorous acid, and flurbiprofen, were excluded from the corresponding pairwise analyses. When a trial included two eligible HA concentrations, the intervention groups were combined using standard formulas for independent groups to avoid duplication of the shared control group. Interventions were classified as multicomponent only when HA was combined with another ingredient intended to exert an independent therapeutic effect, such that the specific effect of HA could not be isolated. Inert vehicles, carriers, and delivery excipients were not considered additional active therapeutic components. Accordingly, polyvinylpyrrolidone was treated as a carrier when it was explicitly described and used solely as a delivery vehicle for HA, as reported by Ceylan Şen et al.; therefore, this trial was eligible for the primary quantitative synthesis. Conversely, formulations containing other potentially active ingredients, or for which the independent contribution of HA could not be distinguished, were excluded from the primary analyses and evaluated only in sensitivity analyses when sufficient data were available. Extracted information was cross-checked against the article text, tables, figures, and available Supplementary Materials. Discrepancies were resolved by re-examination of the original publication and consensus among the reviewers.

The methodological characteristics, intervention protocols, follow-up periods, assessed outcomes, and main findings of the eight included randomized clinical trials are summarized in Table 1.

### 2.6. Risk of Bias Assessment

The risk of bias of the included randomized clinical trials was independently assessed by two reviewers (L.C.H. and B.V.R.) using the revised Cochrane Risk of Bias tool for randomized trials (RoB 2) [23]. Assessments were conducted at the outcome level and focused on the effect of assignment to the intervention. The following five domains were evaluated: bias arising from the randomization process; bias due to deviations from the intended interventions; bias due to missing outcome data; bias in the measurement of the outcome; and bias in the selection of the reported result. The signaling questions and domain-level algorithms provided by RoB 2 were used to classify each domain and the overall result as “low risk of bias,” “some concerns,” or “high risk of bias.” Because risk of bias may differ according to the nature and measurement of each outcome, separate judgments were made when appropriate for complete epithelialization, WHI, postoperative pain, analgesic consumption, bleeding, and color match. Disagreements between reviewers were resolved through discussion and consensus; when consensus could not be reached, a third reviewer (A.O.B.) acted as an adjudicator. The final judgments were summarized using a traffic-light plot.

### 2.7. Assessment of the Certainty of Evidence

The certainty of evidence for each critical outcome was independently evaluated by two reviewers (L.C.H. and B.V.R.) using the Grading of Recommendations Assessment, Development and Evaluation (GRADE) approach [24]. Randomized clinical trial evidence initially received a rating of high certainty and was subsequently considered for downgrading according to five domains: risk of bias, inconsistency, indirectness, imprecision, and publication bias. Certainty was classified as high, moderate, low, or very low. The assessment was conducted separately for complete epithelialization at days 14 and 21, WHI at days 7 and 21, pain VAS at days 3 and 7, analgesic consumption at day 3, postoperative bleeding at days 1 and 3, and color match at days 14 and 21. Inconsistency was judged by considering the direction and magnitude of the study effects together with τ^2^, Cochran’s Q, I^2^, and the corresponding prediction intervals when estimable. Imprecision was assessed according to the width of the confidence intervals, whether they included the null effect or clinically important benefit and harm, and the number of studies, participants, and outcome events. Indirectness was considered in relation to differences in the population, HA formulation, comparator, outcome measurement, and clinical applicability. Publication bias was assessed cautiously because fewer than 10 studies were available for every outcome. Any disagreement was resolved by consensus or consultation with a third reviewer (A.O.B.). The final judgments, reasons for downgrading, relative and absolute effects, and number of participants and studies were presented in a Summary of Findings table.

### 2.8. Statistical Analysis

Separate time-specific meta-analyses were conducted for each prespecified postoperative assessment; therefore, measurements obtained at different time points were not combined within a longitudinal or multilevel model. Dichotomous outcomes, including complete epithelialization and postoperative bleeding, were summarized as risk ratios (RRs), whereas continuous outcomes, including the Wound Healing Index (WHI), pain measured using a visual analogue scale (VAS), analgesic consumption, and color match, were summarized as mean differences (MDs), with corresponding 95% confidence intervals (CIs). Pain scores reported on a 0–100 scale were converted to a common 0–10 scale by dividing the means and standard deviations by 10.

For dichotomous outcomes, study-specific RRs were calculated on the logarithmic scale and pooled using an inverse-variance random-effects model; the Mantel–Haenszel method was not used. When a study contained a zero cell because all participants in one group experienced the outcome, a continuity correction of 0.5 was added symmetrically to all four cells of that study’s 2 × 2 table before calculating the log RR and its sampling variance. The correction was applied only to studies containing a zero cell and was not applied to studies without zero cells. Studies reporting zero events in both groups would have been excluded from relative-effect estimation because they provide no information for calculating an RR; however, no double-zero studies contributed to the risk-ratio meta-analyses. At postoperative day 21, the reported 61/61 value represents the aggregated HA-group data across the three contributing trials (24/24, 15/15, and 22/22), each of which had a zero non-event cell. The same pooling specifications and continuity-correction rule were retained in the corresponding leave-one-out and formulation-based sensitivity analyses.

Random-effects models were fitted using restricted maximum-likelihood (REML) estimation of the between-study variance (τ^2^), and the primary confidence intervals and tests of the overall effect were based on the standard normal distribution (Z). Statistical heterogeneity was evaluated using Cochran’s Q test, τ^2^, and the I^2^ statistic, with I^2^ values of approximately 25%, 50%, and 75% considered to represent low, moderate, and substantial heterogeneity, respectively. When at least three studies were available, 95% prediction intervals were calculated to describe the range in which the effect of a future comparable study might be expected; prediction intervals from analyses containing only two studies were considered unstable and were not interpreted.

Leave-one-out sensitivity analyses were performed for meta-analyses containing three studies by removing one study at a time. Because of the limited number of trials, these analyses were interpreted as exploratory sensitivity analyses rather than definitive tests of robustness. For meta-analyses containing more than two studies and a non-zero REML estimate of τ^2^, Hartung–Knapp-adjusted confidence intervals were calculated as sensitivity analyses while retaining REML for variance estimation. Analyses containing only two studies retained the conventional Z-based method and were considered exploratory. Formulation-based sensitivity analyses compared a primary scenario restricted to interventions in which HA was the sole active therapeutic component, irrespective of the presence of an inert carrier or delivery excipient, with a broader scenario that additionally included products containing other potentially active ingredients whose independent effects could not be isolated.

For complete epithelialization and postoperative bleeding, absolute effects were derived by applying the pooled RR and its 95% CI to the observed control-group risk and were expressed as the estimated number of events with HA per 1000 patients and the absolute risk difference per 1000. The number needed to treat for one additional beneficial outcome (NNTB) was calculated as the reciprocal of the absolute risk difference only when the pooled relative effect was statistically significant; no NNT was calculated when the confidence interval included the null effect. Potential small-study effects were explored graphically using funnel plots when applicable, but no formal statistical assessment of publication bias was undertaken because fewer than 10 studies contributed to every meta-analysis. Statistical significance was defined as a two-sided *p*-value < 0.05; however, interpretation emphasized the magnitude and precision of the effect estimates, heterogeneity, sensitivity analyses, and certainty of evidence rather than statistical significance alone.

## 3. Results

### 3.1. Risk of Bias and Certainty of Evidence

The outcome-specific RoB 2 assessment identified no results at high overall risk of bias. Nabiyi et al. was judged to have a low overall risk of bias for WHI and pain, whereas the results from Yıldırım et al., Lektemur Alpan et al., and Ceylan Şen et al. raised some concerns. These concerns were mainly related to insufficiently described allocation concealment, deviations from the intended interventions, measurement of subjective outcomes, incomplete outcome data, and selection of the reported results. Nevertheless, most individual RoB 2 domains were judged to be at low risk (Figure 2A).

According to GRADE, the certainty of evidence was low for complete epithelialization at days 14 and 21 and for WHI at day 7. Evidence for WHI at day 21, pain at days 3 and 7, analgesic consumption at day 3, postoperative bleeding at days 1 and 3, and color match at days 14 and 21 was rated as very low, primarily because of risk-of-bias concerns, substantial heterogeneity, and imprecision arising from the small number of studies and participants. Despite these limitations, the direction of most pooled estimates favored topical HA. The most consistent favorable findings were observed for complete epithelialization at days 14 and 21 and WHI at day 7, while reductions in analgesic consumption and improvements in color match also suggested potential clinical benefits. Estimates for pain and postoperative bleeding favored HA numerically but remained inconclusive because their confidence intervals included the null effect (Figure 2B).

### 3.2. Complete Epithelialization at Postoperative Day 14

Three randomized clinical trials involving 110 participants were included in the meta-analysis of complete epithelialization at postoperative day 14, comprising 61 participants treated with topical hyaluronic acid (HA) and 49 controls. Complete epithelialization was achieved in 25 participants in the HA groups (41.0%) compared with 10 participants in the control groups (20.4%). The random-effects model showed a statistically significant benefit of topical HA, with treated participants being more than twice as likely to achieve complete epithelialization by day 14 compared with controls (RR = 2.11; 95% CI: 1.13–3.94; *p* = 0.0185; Figure 3). The direction of effect consistently favored HA across all included studies, with no evidence of statistical heterogeneity (τ^2^ = 0; Q = 0.28, df = 2, *p* = 0.8706; I^2^ = 0%). These findings support a beneficial and consistent effect of topical HA on early palatal wound epithelialization. Nevertheless, the 95% prediction interval crossed the null value (0.54–8.30), indicating some uncertainty regarding the magnitude of the effect expected in a future study.

### 3.3. Meta-Analysis of Complete Epithelialization at Postoperative Day 21

Three randomized clinical trials involving 110 participants were included in the meta-analysis of complete epithelialization at postoperative day 21, comprising 61 participants treated with topical hyaluronic acid (HA) and 49 controls. Complete epithelialization was achieved in all participants receiving HA (61/61; 100%) compared with 28 of 49 participants in the control groups (57.1%). The random-effects model demonstrated a significant benefit of topical HA, increasing the probability of complete epithelialization by approximately 62% compared with control treatment (RR = 1.62; 95% CI: 1.29–2.03; *p* < 0.0001; Figure 4). All individual studies showed statistically significant effects favoring HA, supporting the consistency of the observed benefit. Between-study heterogeneity was negligible (τ^2^ = 0.0014; Q = 2.07, df = 2, *p* = 0.3552; I^2^ = 3.4%). These findings provide consistent evidence that topical HA promotes complete palatal wound epithelialization by postoperative day 21. Although the 95% prediction interval marginally crossed the null value (0.96–2.72), its overall direction remained favorable to HA, indicating that uncertainty primarily concerns the magnitude rather than the direction of the treatment effect.

### 3.4. Leave-One-Out Sensitivity Analysis for Complete Epithelialization

Leave-one-out sensitivity analyses were performed for complete epithelialization at postoperative days 14 and 21. At day 14, the overall effect favored topical hyaluronic acid (RR = 2.11; 95% CI: 1.13–3.94). The effect remained statistically significant after excluding Yıldırım et al. (RR = 2.00; 95% CI: 1.04–3.86), but statistical significance was lost after excluding Lektemur Alpan et al. (RR = 2.20; 95% CI: 0.97–4.98) or Ceylan Şen et al. (RR = 2.23; 95% CI: 0.94–5.29). Although all point estimates continued to favor hyaluronic acid, the day-14 finding was sensitive to the exclusion of individual studies. At day 21, the overall effect also favored hyaluronic acid (RR = 1.62; 95% CI: 1.29–2.03), and statistical significance was maintained after excluding Yıldırım et al. (RR = 1.73; 95% CI: 1.21–2.48), Lektemur Alpan et al. (RR = 1.74; 95% CI: 1.21–2.52), or Ceylan Şen et al. (RR = 1.47; 95% CI: 1.14–1.90). Thus, the direction and statistical significance of the day-21 effect were consistent across all sensitivity scenarios. Nevertheless, because each analysis included only three trials, these findings should be interpreted as exploratory sensitivity analyses rather than definitive evidence of robustness (Figure 5).

### 3.5. Meta-Analysis of the Wound Healing Index at Postoperative Days 3, 7, 14, and 21

Three randomized clinical trials involving 113 participants—63 treated with topical hyaluronic acid (HA) and 50 controls—contributed to the time-specific meta-analyses of the Wound Healing Index (WHI). At postoperative day 3, the random-effects model demonstrated significantly higher WHI scores with HA (MD = 0.38; 95% CI: 0.19–0.56; *p* < 0.0001; Figure 6A), with low between-study heterogeneity (τ^2^ = 0.0034; Q = 2.41, df = 2, *p* = 0.2995; I^2^ = 17.1%). At day 7, HA also significantly improved WHI scores (MD = 0.58; 95% CI: 0.42–0.75; *p* < 0.0001; Figure 6B), with no evidence of heterogeneity (τ^2^ = 0; Q = 2.64, df = 2, *p* = 0.267; I^2^ = 0%). A significant benefit was similarly observed at day 14 (MD = 0.42; 95% CI: 0.23–0.60; *p* < 0.0001; Figure 6C), with very low heterogeneity (τ^2^ < 0.0001; Q = 2.19, df = 2, *p* = 0.334; I^2^ = 8.8%). At day 21, the pooled estimate continued to favor HA (MD = 0.53; 95% CI: 0.24–0.82; Figure 6D), and all three individual studies demonstrated statistically significant effects, with mean differences ranging from 0.27 to 0.77 points. At days 3, 7, and 14, all study-specific estimates favored HA, although Nabiyi et al. did not individually reach statistical significance, whereas Lektemur Alpan et al. and Ceylan Şen et al. reported significant improvements. Overall, topical HA improved WHI scores by approximately 0.38–0.58 points on the 1-to-5 scale across the evaluated postoperative assessments, supporting a consistently favorable effect on palatal donor-site healing.

### 3.6. Leave-One-Out Sensitivity Analysis for WHI

Leave-one-out sensitivity analyses were performed for the Wound Healing Index (WHI) at postoperative days 3, 7, 14, and 21 (Figure 7A–D). At day 3, the overall pooled effect favored topical hyaluronic acid (HA) (MD = 0.38; 95% CI: 0.19–0.56), and statistical significance was maintained after excluding Nabiyi et al. (MD = 0.46; 95% CI: 0.25–0.66), Lektemur Alpan et al. (MD = 0.37; 95% CI: 0.03–0.71), or Ceylan Şen et al. (MD = 0.30; 95% CI: 0.10–0.51). Similarly, at day 7, the pooled estimate remained significantly favorable to HA (MD = 0.58; 95% CI: 0.42–0.75), with consistent results after excluding Nabiyi et al. (MD = 0.63; 95% CI: 0.45–0.80), Lektemur Alpan et al. (MD = 0.48; 95% CI: 0.05–0.90), or Ceylan Şen et al. (MD = 0.44; 95% CI: 0.05–0.83).

At day 14, the overall effect significantly favored HA (MD = 0.42; 95% CI: 0.23–0.60). Statistical significance was preserved after excluding Nabiyi et al. (MD = 0.49; 95% CI: 0.28–0.70) or Lektemur Alpan et al. (MD = 0.34; 95% CI: 0.05–0.63). However, exclusion of Ceylan Şen et al. produced a borderline estimate whose confidence interval marginally crossed the null value (MD = 0.36; 95% CI: −0.00 to 0.73). At day 21, the overall effect remained significantly favorable to HA (MD = 0.53; 95% CI: 0.24–0.82), and significance was maintained after excluding Nabiyi et al. (MD = 0.67; 95% CI: 0.47–0.86), Lektemur Alpan et al. (MD = 0.51; 95% CI: 0.02–0.99), or Ceylan Şen et al. (MD = 0.43; 95% CI: 0.11–0.75). Overall, the WHI findings were robust at days 3, 7, and 21, whereas the day-14 result showed marginal sensitivity to the exclusion of Ceylan Şen et al. Given the inclusion of only three studies at each time point, these findings should be interpreted as exploratory sensitivity analyses.

### 3.7. Hartung–Knapp Sensitivity Analysis for the Wound Healing Index

Hartung–Knapp sensitivity analyses were conducted for the Wound Healing Index (WHI) at postoperative days 3 and 21, the time points at which REML estimated a non-zero between-study variance. At day 3, the Z-based reference analysis indicated a statistically significant improvement with topical hyaluronic acid (MD = 0.38; 95% CI: 0.19–0.56). However, the Hartung–Knapp adjustment widened the confidence interval, which crossed the null value (MD = 0.38; 95% CI: −0.04 to 0.79). Similarly, at day 21, the Z-based analysis favored hyaluronic acid (MD = 0.53; 95% CI: 0.24–0.82), whereas the Hartung–Knapp interval included no effect (MD = 0.53; 95% CI: −0.10 to 1.16). Thus, although the magnitude and direction of the pooled estimates remained unchanged, statistical significance was not maintained under Hartung–Knapp. These results indicate that the evidence of improved wound healing at days 3 and 21 is sensitive to the inferential method and should be interpreted cautiously because of the small number of included studies. Hartung–Knapp sensitivity analyses were not performed at days 7 and 14 because REML estimated τ^2^ = 0 at these time points (Figure 8).

### 3.8. Meta-Analysis of Postoperative Pain Assessed Using the VAS at Day 3

At postoperative day 3, three randomized clinical trials involving 119 participants were included, comprising 72 participants treated with topical hyaluronic acid (HA) and 47 controls. The random-effects model showed a reduction in pain favoring HA; however, the pooled effect was not statistically significant (MD = −1.61 points on a 0–10 VAS; 95% CI: −4.52 to 1.30; *p* = 0.2778; Figure 9A). Considerable between-study heterogeneity was observed (τ^2^ = 6.3197; Q = 39.00, df = 2, *p* < 0.0001; I^2^ = 94.9%). Yıldırım et al. reported a substantial and statistically significant reduction in pain, whereas Nabiyi et al. and Ceylan Şen et al. showed small, non-significant differences between groups. Furthermore, the wide prediction interval (−14.18 to 10.95) crossed the null value, indicating substantial uncertainty regarding the magnitude and direction of the effect expected in a future clinical setting. Therefore, although the pooled estimate favored topical HA, the evidence did not demonstrate a consistent reduction in postoperative pain at day 3.

### 3.9. Meta-Analysis of Postoperative Pain Assessed Using the VAS at Day 7

At postoperative day 7, three randomized clinical trials involving 119 participants were included, comprising 72 participants treated with topical hyaluronic acid (HA) and 47 controls. Although the pooled estimate favored HA, the random-effects model did not demonstrate a statistically significant reduction in postoperative pain (MD = −0.97 points on a 0–10 VAS; 95% CI: −3.27 to 1.34; *p* = 0.4095; Figure 9B). Considerable between-study heterogeneity was observed (τ^2^ = 3.8934; Q = 22.37, df = 2, *p* < 0.0001; I^2^ = 91.1%). Yıldırım et al. reported a substantial and statistically significant reduction in pain, whereas Nabiyi et al. and Ceylan Şen et al. found no significant differences between HA and control. The prediction interval was wide and crossed the null value (−10.85 to 8.91), indicating substantial uncertainty regarding the effect expected in a future clinical setting. Thus, despite a numerical trend toward reduced pain, the available evidence did not demonstrate a consistent benefit of topical HA at postoperative day 7.

### 3.10. Leave-One-Out Sensitivity Analysis for Postoperative Pain

Leave-one-out sensitivity analyses were conducted for postoperative pain at days 3 and 7 (Figure 10A,B). At day 3, the overall estimate including all three studies favored topical hyaluronic acid (HA), but was not statistically significant (MD = −1.61; 95% CI: −4.52 to 1.30). Exclusion of Yıldırım et al. markedly attenuated the effect toward the null (MD = −0.13; 95% CI: −0.82 to 0.56). Conversely, the direction of effect remained favorable to HA after excluding Nabiyi et al. (MD = −2.34; 95% CI: −6.78 to 2.10) or Ceylan Şen et al. (MD = −2.39; 95% CI: −6.74 to 1.96), although both confidence intervals were wide and crossed the null value.

A similar pattern was observed at day 7. The overall pooled estimate was not statistically significant (MD = −0.97; 95% CI: −3.27 to 1.34). After excluding Yıldırım et al., the effect approached the null and changed slightly in direction (MD = 0.10; 95% CI: −0.38 to 0.59). Exclusion of Nabiyi et al. (MD = −1.69; 95% CI: −5.03 to 1.65) or Ceylan Şen et al. (MD = −1.49; 95% CI: −5.26 to 2.27) maintained a numerical effect favoring HA, but neither scenario reached statistical significance. Overall, no leave-one-out scenario produced a statistically significant effect. These exploratory sensitivity analyses indicate that Yıldırım et al. substantially influenced the magnitude and direction of the pooled estimates, demonstrating that the findings for postoperative pain were not robust to the exclusion of individual studies.

### 3.11. Hartung–Knapp Sensitivity Analysis for Pain VAS

Hartung–Knapp sensitivity analyses were performed for pain VAS at postoperative days 3 and 7 while retaining REML estimation of the between-study variance. At day 3, the conventional Z-based analysis yielded a pooled mean difference of −1.61 (95% CI: −4.52 to 1.30), whereas the Hartung–Knapp adjustment produced a substantially wider confidence interval (MD = −1.61; 95% CI: −8.04 to 4.81). Similarly, at day 7, the pooled estimate remained −0.97, but the confidence interval widened from −3.27 to 1.34 under the Z-based approach to −6.11 to 4.18 with the Hartung–Knapp adjustment. In both analyses, the point estimates favored topical hyaluronic acid, but the confidence intervals crossed the null value irrespective of the inferential method. Therefore, the Hartung–Knapp adjustment did not alter the statistical conclusion, although it substantially increased uncertainty because of the small number of studies and considerable between-study heterogeneity. These findings should consequently be interpreted as exploratory (Figure 11A,B).

### 3.12. Meta-Analysis of Analgesic Consumption at Postoperative Day 1

At postoperative day 1, two randomized clinical trials involving 74 participants were included, with 37 participants treated with topical hyaluronic acid (HA) and 37 controls. Both studies recorded the daily number of 500-mg paracetamol tablets. The random-effects model showed lower analgesic consumption in the HA groups; however, the pooled effect was not statistically significant (MD = −0.60 tablets; 95% CI: −1.73 to 0.52; *p* = 0.2929; Figure 12A). Considerable between-study heterogeneity was observed (τ^2^ = 0.6007; Q = 10.92, df = 1, *p* = 0.0009; I^2^ = 90.8%). Lektemur Alpan et al. reported a significant reduction in analgesic consumption, whereas Ceylan Şen et al. found virtually no difference between HA and control. Because only two studies were available, the prediction interval was considered unstable and was not interpreted. Overall, the evidence did not demonstrate a consistent reduction in analgesic consumption with topical HA during the first postoperative day.

### 3.13. Meta-Analysis of Analgesic Consumption at Postoperative Day 3

At postoperative day 3, two randomized clinical trials involving 74 participants were included, with 37 participants treated with topical hyaluronic acid (HA) and 37 controls. Both studies recorded the daily number of 500-mg paracetamol tablets. The random-effects model demonstrated significantly lower analgesic consumption in the HA groups than in the control groups (MD = −0.80 tablets; 95% CI: −1.34 to −0.26; *p* = 0.0035; Figure 12B). Both individual studies showed statistically significant effects favoring HA, with reductions ranging from 0.55 to 1.10 tablets. Moderate-to-substantial between-study heterogeneity was observed (τ^2^ = 0.1028; Q = 3.12, df = 1, *p* = 0.0772; I^2^ = 68.0%). Because only two studies were available, the prediction interval was considered unstable and was not interpreted. Overall, topical HA was associated with an average reduction of 0.80 paracetamol tablets during the third postoperative day, although the limited number of studies warrants cautious interpretation.

### 3.14. Meta-Analysis of Postoperative Bleeding at Day 1

At postoperative day 1, two randomized clinical trials involving 74 participants were included, with 37 participants treated with topical hyaluronic acid (HA) and 37 controls. Postoperative bleeding occurred in 12 participants in the HA groups (32.4%) and 17 participants in the control groups (45.9%). The random-effects model showed a lower risk of bleeding with HA; however, the pooled effect was not statistically significant (RR = 0.72; 95% CI: 0.37–1.40; *p* = 0.3362; Figure 13A). Between-study heterogeneity was low (τ^2^ = 0.0437; Q = 1.19, df = 1, *p* = 0.2756; I^2^ = 15.9%). Although the pooled estimate corresponded to a 28% relative reduction in bleeding risk, the confidence interval crossed the null value. Because only two studies were available, the prediction interval was considered unstable and was not interpreted. Overall, the evidence suggested a possible reduction in early postoperative bleeding with topical HA, but the available data were insufficient to establish a statistically significant effect.

### 3.15. Meta-Analysis of Postoperative Bleeding at Day 3

At postoperative day 3, two randomized clinical trials involving 74 participants were included, with 37 participants treated with topical hyaluronic acid (HA) and 37 controls. Postoperative bleeding occurred in 2 participants in the HA groups (5.4%) and 6 participants in the control groups (16.2%). The random-effects model showed a lower risk of bleeding with HA; however, the pooled effect did not reach statistical significance (RR = 0.36; 95% CI: 0.07–1.80; *p* = 0.2120; Figure 13B). No between-study heterogeneity was detected (τ^2^ = 0; Q = 0.87, df = 1, *p* = 0.3508; I^2^ = 0%). Although the pooled estimate corresponded to a 64% relative reduction in bleeding risk, the confidence interval was wide and crossed the null value, reflecting the small number of events and limited sample size. Because only two studies were included, the prediction interval was considered unstable and was not interpreted. Overall, topical HA showed a potentially beneficial effect on postoperative bleeding at day 3, but the available evidence remained inconclusive.

### 3.16. Meta-Analysis of Palatal Tissue Color Match at Postoperative Day 14

At postoperative day 14, three randomized clinical trials involving 110 participants were included, comprising 61 participants treated with topical hyaluronic acid (HA) and 49 controls. Color-match scores were harmonized to a 0–10 scale, with higher values indicating better color correspondence with the surrounding palatal tissue. The random-effects model demonstrated significantly higher color-match scores in the HA groups than in the control groups (MD = 1.43 points; 95% CI: 0.67–2.19; *p* = 0.0002; Figure 14A). Yıldırım et al. and Lektemur Alpan et al. reported statistically significant improvements, whereas the estimate from Ceylan Şen et al. favored HA but did not individually reach statistical significance. Substantial between-study heterogeneity was observed (τ^2^ = 0.2984; Q = 6.61, df = 2, *p* = 0.0367; I^2^ = 69.8%). Furthermore, the prediction interval crossed the null value (−1.45 to 4.31), indicating uncertainty regarding the effect expected in a future clinical setting. Overall, topical HA was associated with improved palatal tissue color matching at postoperative day 14, although the magnitude of benefit varied across studies.

### 3.17. Meta-Analysis of Palatal Tissue Color Match at Postoperative Day 21

At postoperative day 21, three randomized clinical trials involving 110 participants were included, comprising 61 participants treated with topical hyaluronic acid (HA) and 49 controls. Color-match scores were harmonized to a 0–10 scale, with higher values indicating better correspondence with the surrounding palatal tissue. The random-effects model demonstrated significantly higher color-match scores in the HA groups than in the control groups (MD = 1.33 points; 95% CI: 0.43–2.22; *p* = 0.0036; Figure 14B). All three studies reported statistically significant effects favoring HA, with mean differences ranging from 0.64 to 2.21 points. Nevertheless, considerable between-study heterogeneity was observed (τ^2^ = 0.5407; Q = 14.58, df = 2, *p* = 0.0007; I^2^ = 86.3%), indicating substantial variation in the magnitude of benefit. The prediction interval crossed the null value (−2.40 to 5.05), reflecting uncertainty regarding the effect expected in a future clinical setting. Overall, topical HA was associated with improved palatal tissue color matching at postoperative day 21, although the magnitude of this improvement differed considerably among studies.

### 3.18. Leave-One-Out Sensitivity Analysis for Color Match

Leave-one-out sensitivity analyses were performed to examine the influence of individual studies on color-match outcomes at postoperative days 14 and 21 (Figure 15A,B). At day 14, the overall pooled effect significantly favored topical hyaluronic acid (HA) (MD = 1.43; 95% CI: 0.67–2.19). The effect remained statistically significant after excluding Yıldırım et al. (MD = 1.36; 95% CI: 0.18–2.54), Lektemur Alpan et al. (MD = 1.08; 95% CI: 0.26–1.91), or Ceylan Şen et al. (MD = 1.85; 95% CI: 1.37–2.34). These findings indicate that the improvement in color matching at day 14 was robust to the exclusion of any individual study.

At day 21, the overall pooled estimate also significantly favored HA (MD = 1.33; 95% CI: 0.43–2.22). Statistical significance was maintained after excluding Yıldırım et al. (MD = 0.90; 95% CI: 0.35–1.45) or Ceylan Şen et al. (MD = 1.69; 95% CI: 0.70–2.68). However, exclusion of Lektemur Alpan et al. produced a wider confidence interval that crossed the null value (MD = 1.41; 95% CI: −0.14 to 2.95). Thus, although all sensitivity estimates remained directionally favorable to HA, the statistical robustness of the day-21 result was partially dependent on the inclusion of Lektemur Alpan et al. Given that only three studies were available, these findings should be interpreted as exploratory sensitivity analyses.

### 3.19. Hartung–Knapp Sensitivity Analysis for Color Match

Hartung–Knapp sensitivity analyses were conducted for color match at postoperative days 14 and 21 while retaining REML estimation of the between-study variance. At day 14, the Z-based reference analysis indicated a statistically significant benefit of topical hyaluronic acid (MD = 1.43; 95% CI: 0.67–2.19). However, applying the Hartung–Knapp adjustment substantially widened the confidence interval, which crossed the null value (MD = 1.43; 95% CI: −0.18 to 3.04). A similar pattern was observed at day 21: the Z-based analysis favored hyaluronic acid (MD = 1.33; 95% CI: 0.43–2.22), whereas the Hartung–Knapp interval included no effect (MD = 1.33; 95% CI: −0.64 to 3.29). Thus, although the direction and magnitude of the pooled estimates remained unchanged, statistical significance was not maintained after the Hartung–Knapp adjustment. These findings indicate that the evidence of improved color match is sensitive to the inferential method and should be interpreted cautiously because of the small number of studies and substantial between-study heterogeneity (Figure 16).

### 3.20. Sensitivity Analysis According to HA Formulation

Formulation-based sensitivity analyses were feasible for pain VAS at day 3 and postoperative bleeding at days 1 and 3. For pain at day 3, the analysis restricted to formulations containing HA as the sole active therapeutic component yielded an MD of −1.61 (95% CI: −4.52 to 1.30). After including the multicomponent HA-containing product, the pooled estimate remained nearly unchanged (MD: −1.59; 95% CI: −3.65 to 0.47). For postoperative bleeding at day 1, the RR changed from 0.72 (95% CI: 0.37 to 1.40) in the principal analysis to 0.42 (95% CI: 0.14 to 1.23) after inclusion of the multicomponent formulation. Similarly, at day 3, the RR changed from 0.36 (95% CI: 0.07 to 1.80) to 0.29 (95% CI: 0.07 to 1.18). Although the inclusion of multicomponent HA-containing products shifted the bleeding estimates further toward a potential benefit, all confidence intervals included the null value. Therefore, formulation-related sensitivity analyses did not materially alter the qualitative interpretation of the findings. Given the small number of studies and the addition of only one multicomponent-product trial per outcome, these results should be considered exploratory (Figure 17).

### 3.21. Absolute Effects

For complete epithelialization at day 14, the baseline risk in the control group was 204 per 1000 patients. Based on the pooled relative effect, the estimated risk with topical HA was 431 per 1000 (95% CI: 231 to 804), corresponding to 227 additional patients achieving complete epithelialization per 1000 treated (95% CI: 27 to 600). The number needed to treat for one additional beneficial outcome (NNTB) was 5. At day 21, the control risk was 571 per 1000, compared with an estimated 926 per 1000 with HA (95% CI: 737 to 1000), representing 354 additional complete epithelialization events per 1000 treated (95% CI: 166 to 429) and an NNTB of 3.

For postoperative bleeding, the control risk at day 1 was 459 per 1000 patients, whereas the estimated risk with HA was 331 per 1000 (95% CI: 170 to 643), corresponding to 129 fewer bleeding events per 1000 treated (95% CI: 289 fewer to 184 more). At day 3, the control risk was 162 per 1000, compared with an estimated risk of 58 per 1000 with HA (95% CI: 11 to 292), corresponding to 104 fewer bleeding events per 1000 treated (95% CI: 151 fewer to 130 more). Because the confidence intervals for bleeding included both a potential benefit and potential harm, an NNT was not calculated (Figure 18).

## 4. Discussion

The findings of this review suggest that the main clinical effect of topical hyaluronic acid (HA) following autogenous gingival graft harvesting is primarily related to palatal wound healing rather than to a consistent reduction in postoperative morbidity. Complete epithelialization represents the most consistent signal: the benefit observed at day 14 was clinically meaningful, although still dependent on individual studies, whereas at day 21 the direction of effect remained stable across all sensitivity analyses. This temporal distinction is relevant because a statistically significant association does not necessarily imply that the effect will be equally reproducible across different clinical settings. Indeed, the prediction intervals crossed or approached the null value despite the low statistical heterogeneity, warranting a cautious interpretation of the pooled estimates. From a clinical perspective, however, the absolute effects are potentially relevant: approximately 227 additional patients per 1000 would achieve complete epithelialization by day 14 and 354 additional patients per 1000 by day 21, corresponding to NNTBs of 5 and 3, respectively. This potential acceleration of epithelial coverage is particularly relevant for palatal wounds healing by secondary intention and is consistent with recent reviews identifying healing rate, pain, and bleeding complications as key components of donor-site morbidity [25,26].

The accelerated epithelialization also has a plausible biological basis. HA does not act merely as a hydrating coating but as a functional component of the extracellular matrix capable of modulating cell migration and proliferation, inflammatory signaling, and the organization of the wound-healing microenvironment. Experimental studies have shown that specific HA molecular weights and concentrations promote keratinocyte migration and increase the expression of mediators involved in angiogenesis and tissue remodeling, including vascular endothelial growth factor (VEGF) and matrix metalloproteinases. In parallel, the immune response to HA depends on its molecular size, with high- and low-molecular-weight fractions exerting different effects on inflammation and tissue repair [27,28]. These mechanisms provide a reasonable explanation for the greater likelihood of epithelialization observed in this review, but they also illustrate why “hyaluronic acid” should not be regarded as a single biologically uniform intervention. Concentration, molecular weight, viscosity, delivery vehicle, wound-retention time, and application frequency may alter tissue exposure and probably contribute to the differences among the included trials.

The observed changes in the Wound Healing Index (WHI) complement this interpretation, although they require greater caution. Mean differences of approximately 0.4–0.6 points on a five-point scale across the assessed time points indicate improved clinical wound healing. Nevertheless, their clinical relevance is more difficult to establish because no validated minimal clinically important difference is available for this outcome in palatal wounds. Furthermore, although the leave-one-out analyses were generally stable, statistical significance at certain time points was not maintained when the Hartung–Knapp adjustment was applied. This indicates that the direction of effect was relatively consistent, but its precision was constrained by the availability of only three studies. A similar pattern was observed for color match: the pooled differences favored HA at days 14 and 21, but the high heterogeneity and loss of statistical significance after Hartung–Knapp adjustment preclude the assumption of a uniform esthetic effect. Evidence from other periodontal settings follows a comparable pattern. Reviews across different therapeutic contexts have reported favorable effects of HA on healing parameters but have also highlighted substantial variability in formulations, treatment protocols, and effect magnitude. More recent analyses have shown that some associations are no longer conclusive when restricted to methodologically more robust evidence [29,30].

Patient-perceived morbidity followed a different pattern from that of the clinical healing outcomes. Although the pooled pain estimates at days 3 and 7 favored HA, the confidence intervals were wide, the prediction intervals encompassed both benefit and no effect, and heterogeneity exceeded 90%. The leave-one-out analysis also demonstrated that one study substantially influenced the magnitude of the effect. Therefore, these findings do not support the conclusion that HA consistently reduces postoperative pain. The reduction in paracetamol consumption at day 3—approximately 0.8 fewer 500-mg tablets—is a favorable signal, but it was derived from only two trials and was accompanied by considerable heterogeneity. Statistical significance alone does not guarantee a perceptible benefit for all patients. The discrepancy between self-reported pain and analgesic consumption is not unexpected: although these outcomes are related, they capture different dimensions and are influenced by individual preferences, expectations, medication instructions, and the characteristics of the mechanical protection applied to the wound. The literature examining other strategies for managing palatal donor sites similarly indicates that clinical healing, pain, and analgesic consumption are related but not necessarily equivalent dimensions of postoperative recovery [31,32].

The potential effect on bleeding warrants an equally cautious interpretation. The RRs of 0.72 on day 1 and 0.36 on day 3 suggest potentially relevant relative reductions; however, the confidence intervals were compatible with both benefit and no effect, and the estimates were based on a small number of events. The absolute estimates further underscore this uncertainty: although 129 fewer bleeding episodes per 1000 patients were projected at day 1 and 104 fewer per 1000 at day 3, the corresponding intervals also allowed for a possible increase in bleeding. Therefore, calculating an NNT was not appropriate. The formulation-based sensitivity analysis shifted the estimates toward greater benefit when multicomponent products were included, but it did not alter the statistical conclusion. This pattern is clinically consistent with the multifactorial nature of palatal hemostasis, which depends on graft depth and extent, vascular anatomy, compression, covering materials, and surgical management, in addition to the topical agent used. Studies evaluating other local strategies at palatal donor sites have likewise treated bleeding, pain, epithelialization, and clinical healing characteristics as distinct outcomes, supporting the need to assess each dimension of recovery independently [33,34].

A particularly important feature of this review is that its interpretation was not based solely on *p*-values. The sensitivity analyses revealed different levels of stability: the effect on epithelialization at day 21 was comparatively robust; the effect at day 14 was more dependent on individual studies; the WHI consistently favored HA but became less precise in some Hartung–Knapp analyses; and pain and color match showed considerable uncertainty. Prediction intervals are especially informative in this context because several crossed the null value even when the 95% CI for the average effect indicated statistical significance. This means that the observed average effect may coexist with a less favorable response, no effect, or an effect of substantially different magnitude in a future comparable trial. Such caution is particularly necessary when only three studies contribute to each synthesis, as both the between-study variance and the prediction intervals themselves are imprecisely estimated. Similarly, the widening of the confidence intervals after Hartung–Knapp adjustment does not necessarily contradict the primary analyses; rather, it more explicitly reflects the uncertainty arising from the small number of studies [35,36].

Clinical heterogeneity is likely one of the main limitations of the available evidence. The trials differed in HA concentration and characteristics, delivery vehicles and systems, the number and frequency of applications, treatment duration, the use of periodontal dressings, sponges, stents, or chlorhexidine, and the characteristics of the control groups. Moreover, some of the evidence was derived from products containing other active components, meaning that their effects cannot be attributed exclusively to HA. Accordingly, these studies were included only in sensitivity analyses whenever possible. Differences also existed in the definitions and measurement methods used for epithelialization, WHI, pain, and tissue color. Therefore, the absence of statistical heterogeneity in some meta-analyses should not be interpreted as evidence of clinical homogeneity, particularly when only two or three studies were available. From a methodological perspective, small meta-analyses are especially sensitive to the heterogeneity estimator, the method used to construct the intervals, and individual studies exerting disproportionate influence. This issue has also been specifically documented in the oral-health meta-analysis literature [37,38].

An additional source of clinical heterogeneity was the variation in HA concentration across the included trials, including formulations containing approximately 0.2%, 0.6%, and 0.8% HA. Accordingly, the pooled estimates should be interpreted as average effects of the evaluated topical HA interventions rather than as concentration-specific treatment effects. Although the random-effects models allowed the underlying effects to vary across studies, they cannot eliminate or fully explain heterogeneity resulting from differences in concentration, molecular weight, viscosity, delivery vehicle, application frequency, treatment duration, and concomitant wound protection. Concentration-specific subgroup analysis or meta-regression was not considered reliable because only two or three studies contributed to each outcome and postoperative assessment, and HA concentration was closely associated with other formulation and protocol characteristics. Consequently, the present evidence cannot establish a dose–response relationship or identify an optimal HA concentration. Directly comparative randomized trials in which concentration is varied while the formulation, delivery system, and application protocol are held constant are required to address this question.

The dosage form and delivery system may also influence the apparent effect of HA on palatal epithelialization. Conventional gels are easy to apply and can provide hydration and direct wound-surface exposure, but their residence time may be limited by saliva, swallowing, tongue movement, and oral function, particularly when they are applied without mechanical protection. Mucoadhesive preparations may prolong retention and maintain closer contact between HA and the wound surface, potentially increasing local exposure and reducing the need for frequent reapplication. Impregnated gelatin or collagen sponges may function as local reservoirs that facilitate gradual HA release while also stabilizing the blood clot, maintaining a moist environment, and providing mechanical wound protection. Similarly, periodontal dressings and acrylic stents may independently reduce mechanical irritation and protect the wound, making it difficult to separate the pharmacological effect of HA from the effect of the delivery system. Differences in retention time, release kinetics, wound coverage, reapplication frequency, and mechanical protection could therefore contribute to variation in epithelialization outcomes. Because dosage form was closely associated with HA concentration, application protocol, and comparator type, and because only a few studies evaluated each delivery system, the available evidence does not permit a reliable comparison of gels, mucoadhesives, and impregnated sponges or identification of the optimal delivery method.

The strengths of this review include the time-specific synthesis of postoperative outcomes, which avoided combining biologically distinct phases of wound healing; the use of random-effects models with restricted maximum likelihood estimation; the joint consideration of τ^2^, I^2^, and prediction intervals; leave-one-out and Hartung–Knapp sensitivity analyses; sensitivity analyses according to formulation composition; the presentation of absolute effects and NNTBs when appropriate; and the assessment of risk of bias and certainty of evidence using RoB 2 and GRADE. Nevertheless, these strengths do not overcome the small number of trials and participants, the limited number of events for some outcomes, the clinical heterogeneity of the interventions, and the imprecision of several estimates. Reliable assessments of small-study effects were also not possible. When inspected, funnel plots should therefore be regarded as exploratory only and cannot exclude publication bias given the small number of studies. Visual or statistical assessments of funnel-plot asymmetry have limited reliability in small meta-analyses and may be affected by heterogeneity, methodological differences, and factors other than publication bias [39,40].

Although eight randomized trials were included in the systematic review, this number should not be interpreted as providing conclusive evidence. The trials evaluated different outcomes, postoperative assessments, HA formulations, application protocols, and comparators; consequently, only two or three studies contributed to each time-specific meta-analysis. The effective evidence base for each pooled estimate was therefore considerably smaller than the total number of included trials. Moreover, several analyses were characterized by small sample sizes, few outcome events, substantial heterogeneity, wide confidence or prediction intervals, and sensitivity to the inferential method or exclusion of individual studies. Accordingly, the certainty of evidence ranged from low to very low. The current meta-analysis identifies clinically relevant signals—particularly for complete epithelialization—but does not establish definitive efficacy across all healing and morbidity outcomes. The findings should therefore be regarded as provisional and hypothesis-strengthening rather than conclusive.

Overall, the available evidence supports considering topical HA a promising adjunct for palatal donor-site healing, with a particularly consistent signal toward earlier epithelialization and favorable findings for certain clinical parameters. However, the current evidence does not demonstrate with equal certainty that HA reduces pain or bleeding, nor does it establish which formulation, concentration, or application regimen provides the greatest benefit. Future research should prioritize multicenter randomized trials with sample sizes calculated for clinically relevant outcomes; chemically well-characterized HA formulations, including their concentration, molecular weight, and additional components; comparator groups receiving equivalent mechanical wound protection; standardized definitions of epithelialization and wound-healing scales; blinded outcome assessment whenever feasible; and prospectively harmonized follow-up time points. In addition to statistical significance, minimal clinically important difference thresholds should be established for continuous outcomes, and patient-centered outcomes, including quality of life, oral function, and satisfaction, should be systematically incorporated. Until more robust evidence becomes available, HA may be considered a complementary option for promoting palatal wound healing, but it should not be regarded as an intervention with uniformly demonstrated benefits across all components of postoperative morbidity.

## 5. Conclusions

Topical hyaluronic acid may promote palatal donor-site healing after autogenous gingival graft harvesting, particularly by increasing complete epithelialization at postoperative days 14 and 21. Favorable time-specific effects were also observed for the Wound Healing Index, analgesic consumption, and tissue color match. However, the findings for WHI and color match were sensitive to Hartung–Knapp adjustment, while the effects on postoperative pain and bleeding remained inconclusive. Although no outcome was classified as having a high overall risk of bias, the certainty of evidence ranged from low to very low because of the limited number of trials and participants, imprecision, and heterogeneity. Therefore, topical hyaluronic acid appears to be a promising adjunct for improving palatal wound healing, but the magnitude and consistency of its clinical benefits remain uncertain. Larger, adequately powered randomized clinical trials using standardized HA formulations, application protocols, outcome definitions, and follow-up schedules are required to confirm these findings.

## Figures and Tables

**Figure 1:**
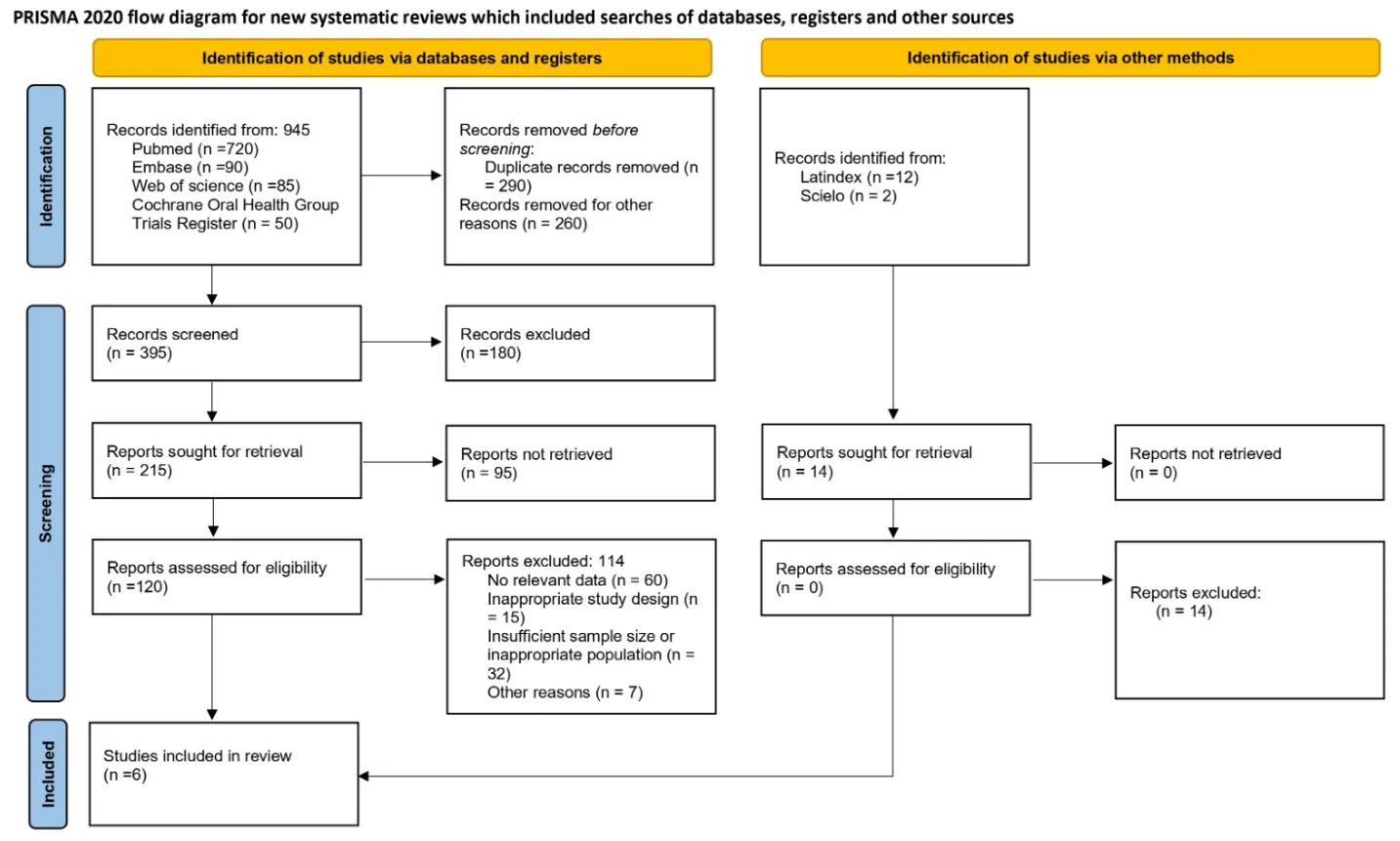
Flowchart of selected studies.

**Figure 2:**
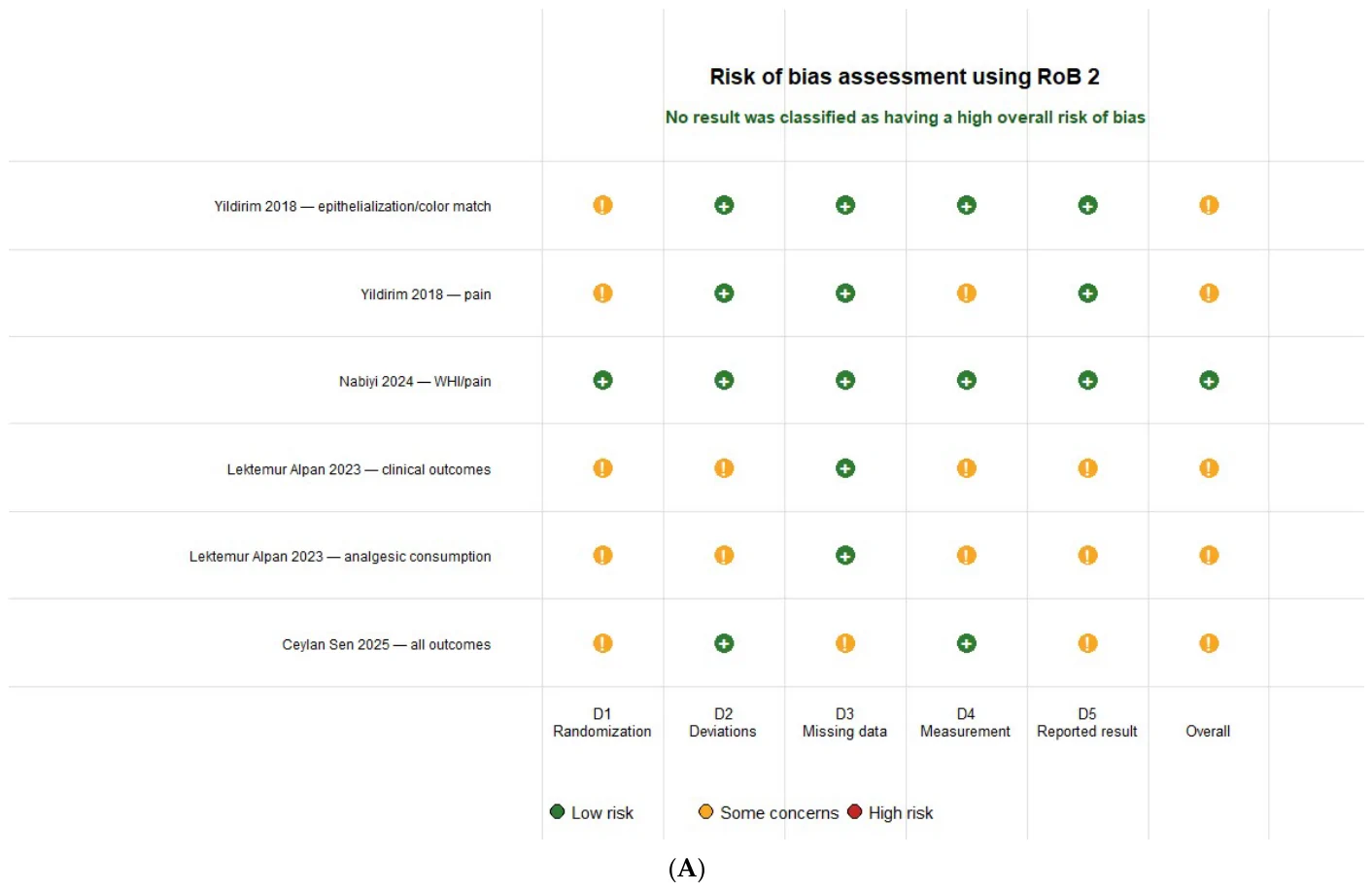

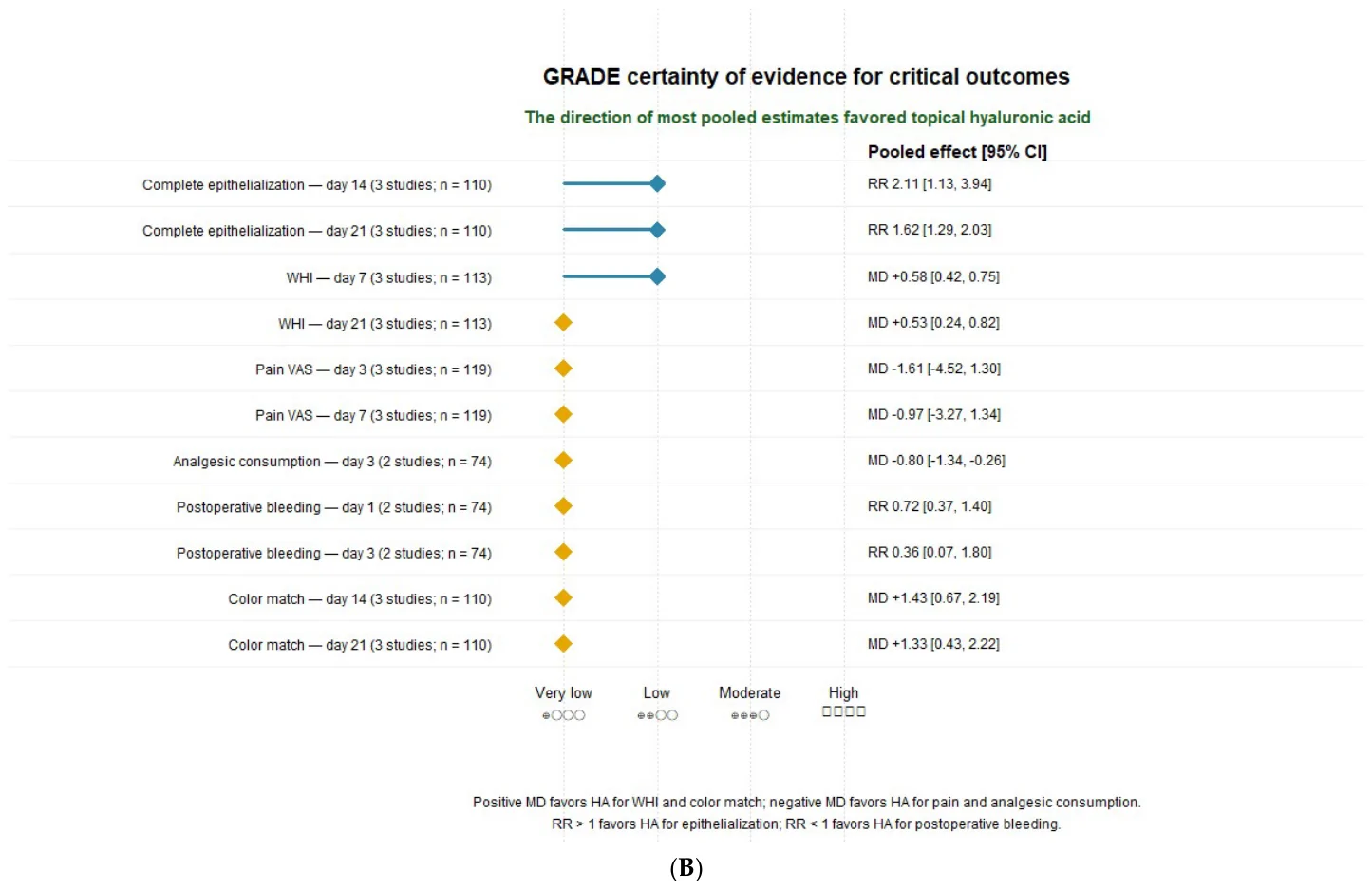
Risk of bias and certainty of evidence. (**A**) Outcome-specific RoB 2 assessment of the included randomized clinical trials. Green indicates low risk of bias, yellow indicates some concerns, and red indicates high risk of bias. (**B**) GRADE certainty ratings and pooled effect estimates with 95% confidence intervals for the prespecified critical outcomes. Blue diamonds indicate low-certainty evidence, whereas yellow diamonds indicate very-low-certainty evidence. CI, confidence interval; GRADE, Grading of Recommendations Assessment, Development and Evaluation; HA, hyaluronic acid; MD, mean difference; RoB 2, revised Cochrane Risk of Bias tool; RR, risk ratio; VAS, visual analogue scale; WHI, Wound Healing Index [10,11,12,13,15,16,17,22].

**Figure 3:**
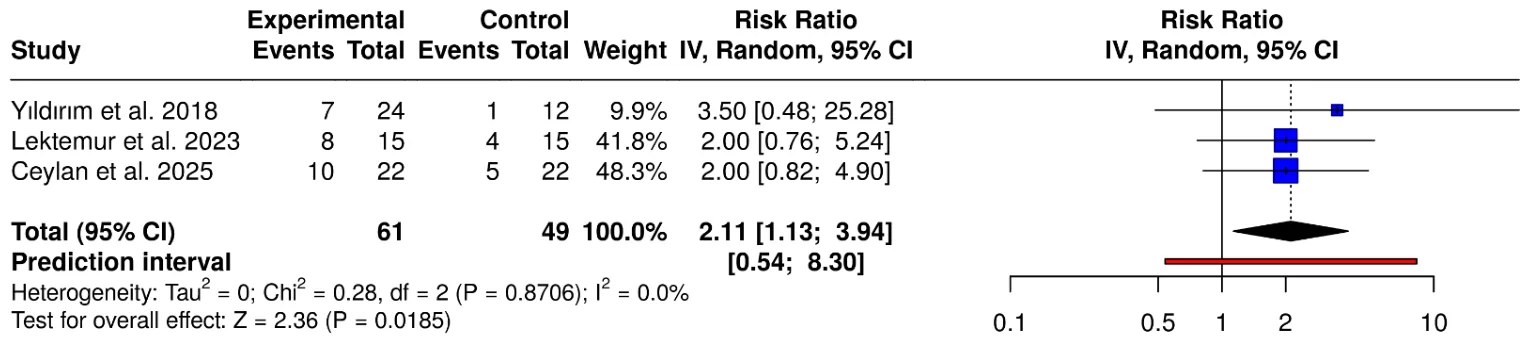
Effect of topical hyaluronic acid on complete epithelialization at postoperative day 14 [10,13,17].

**Figure 4:**
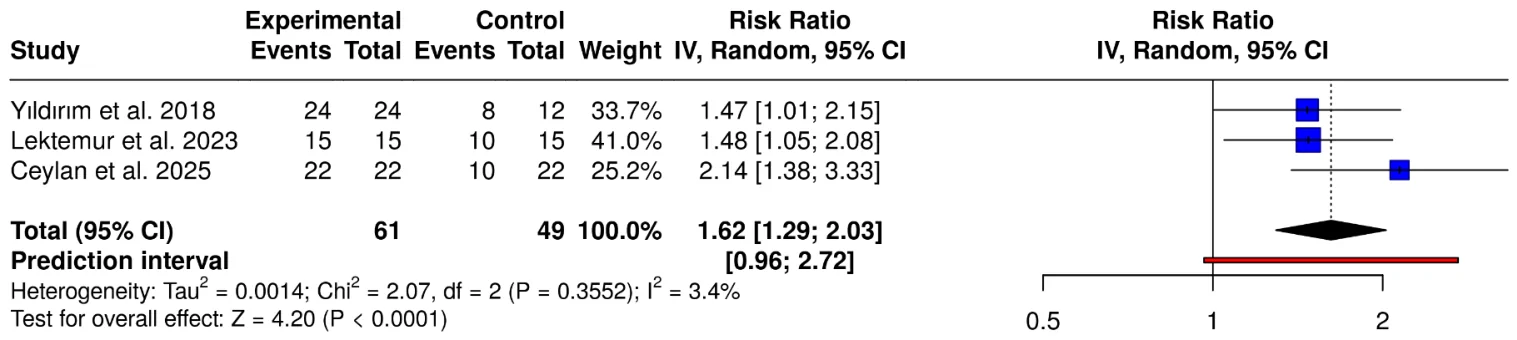
Effect of topical hyaluronic acid on complete epithelialization at postoperative day 21 [10,13,17].

**Figure 5:**
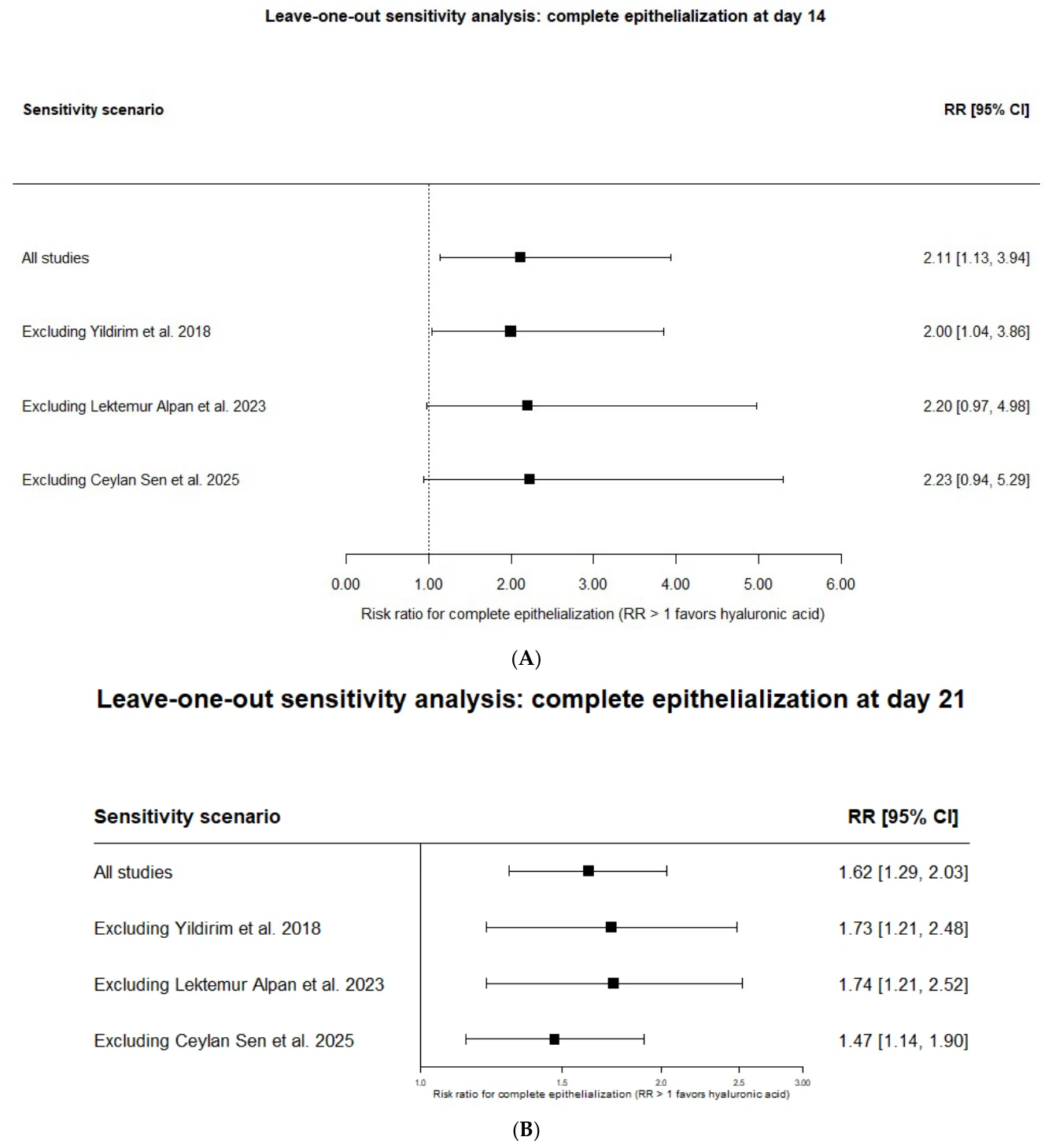
**Leave-one-out sensitivity analyses for complete epithelialization.** (**A**) Sensitivity analysis at postoperative day 14. (**B**) Sensitivity analysis at postoperative day 21. Each analysis shows the pooled risk ratio after sequential exclusion of each individual study. Risk ratios greater than 1 favor topical hyaluronic acid. CI, confidence interval; HA, hyaluronic acid; RR, risk ratio [10,13,17].

**Figure 6:**
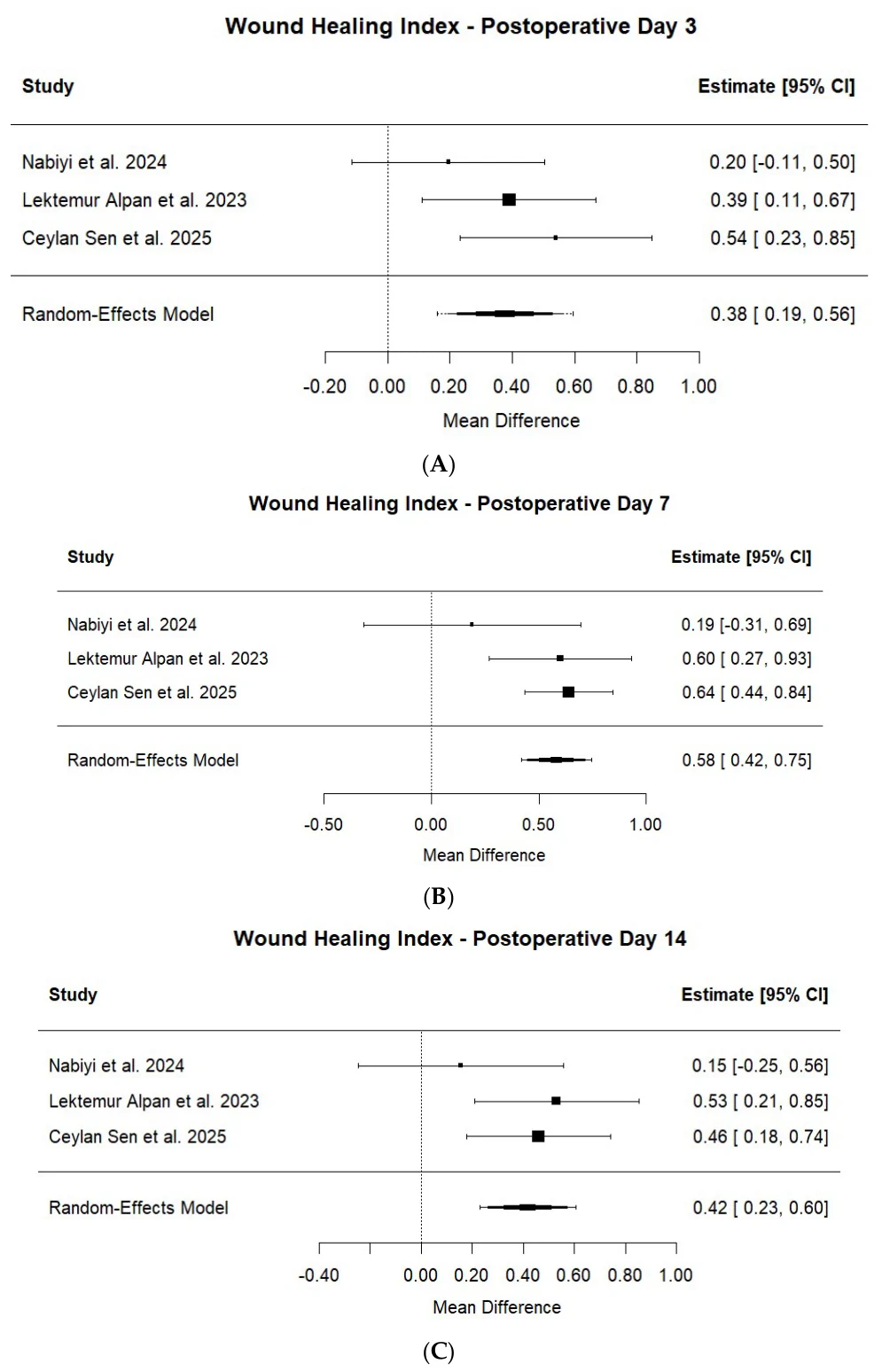

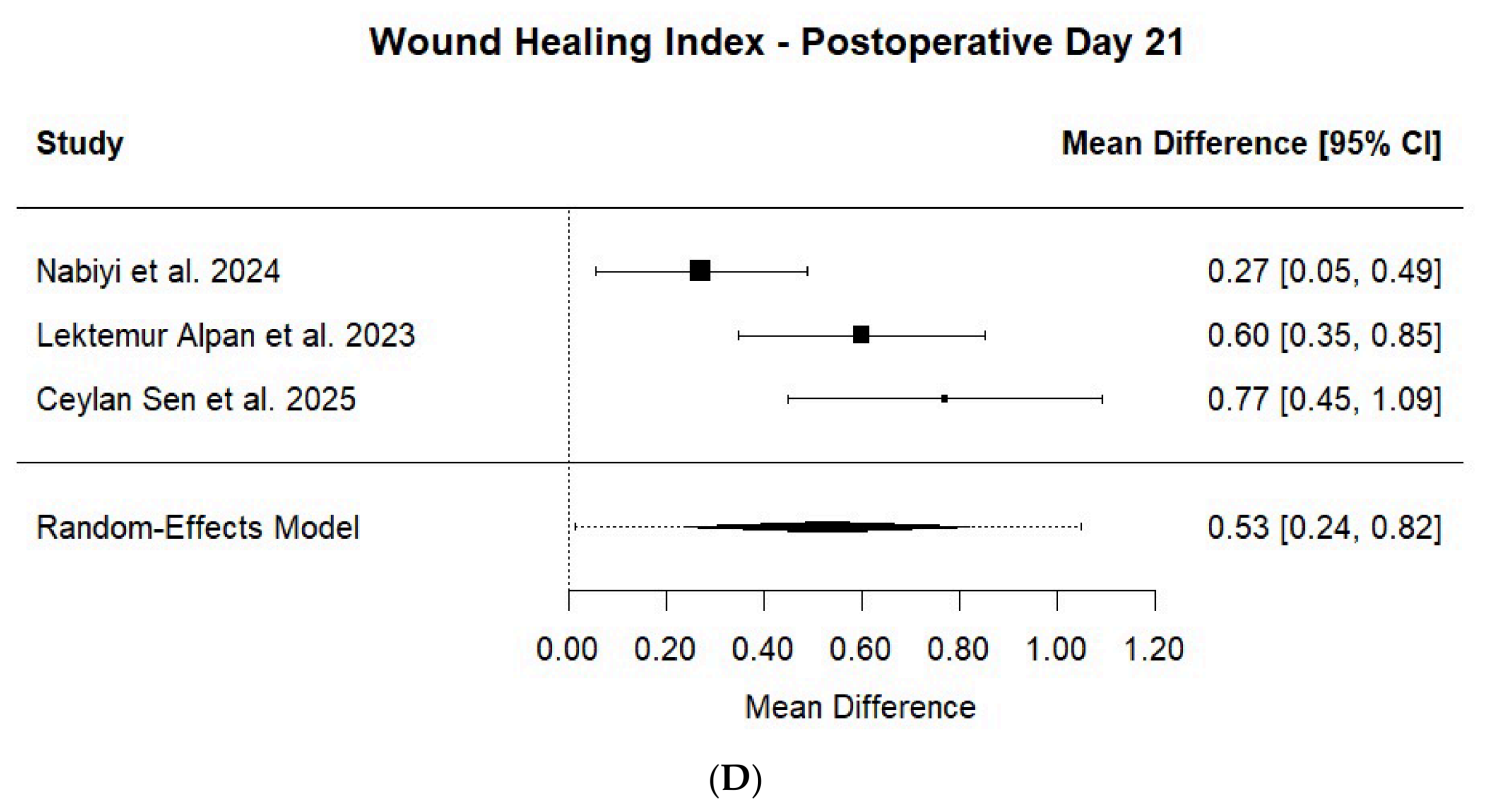
Effects of topical hyaluronic acid on the Wound Healing Index at postoperative (**A**) day 3, (**B**) day 7, (**C**) day 14, and (**D**) day 21. Forest plots present pooled mean differences and corresponding 95% confidence intervals calculated using random-effects models. Positive mean differences favor topical hyaluronic acid. CI, confidence interval; HA, hyaluronic acid; MD, mean difference; WHI, Wound Healing Index [13,17,22].

**Figure 7:**
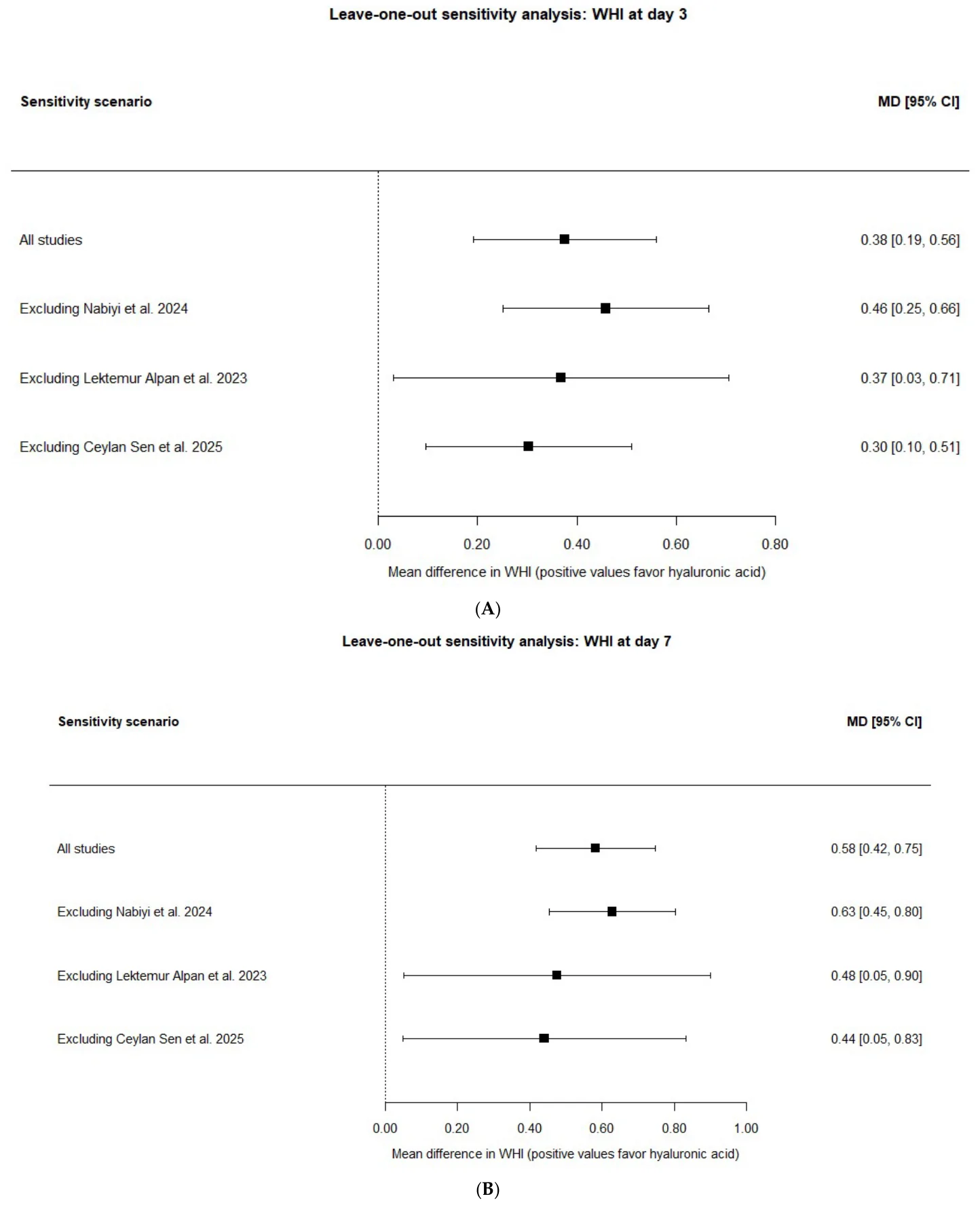

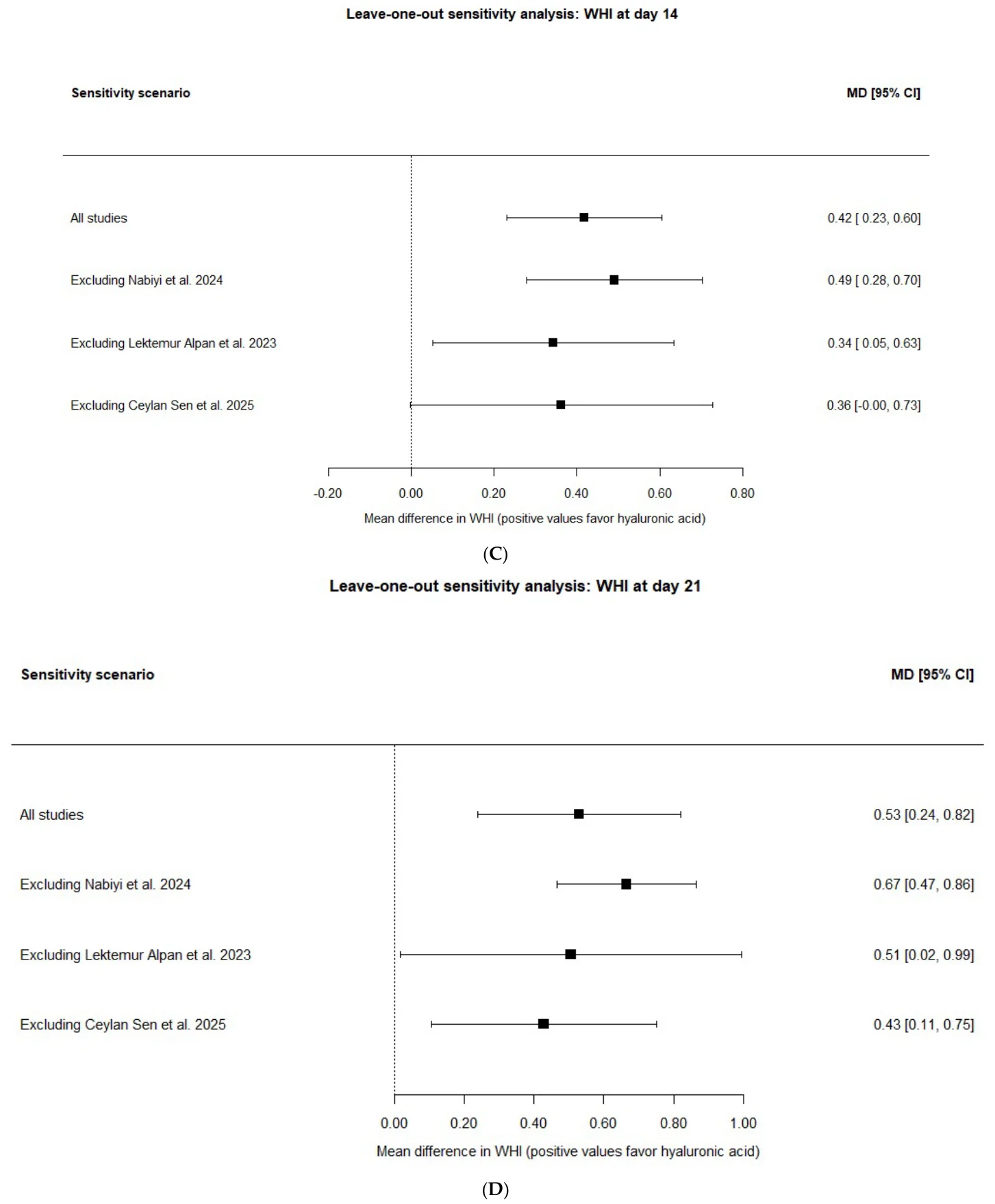
**Leave-one-out sensitivity analyses for the Wound Healing Index.** (**A**) Postoperative day 3. (**B**) Postoperative day 7. (**C**) Postoperative day 14. (**D**) Postoperative day 21. Each analysis presents the pooled mean difference after sequential exclusion of each individual study. Positive mean differences favor topical hyaluronic acid. CI, confidence interval; HA, hyaluronic acid; MD, mean difference; WHI, Wound Healing Index [13,17,22].

**Figure 8:**
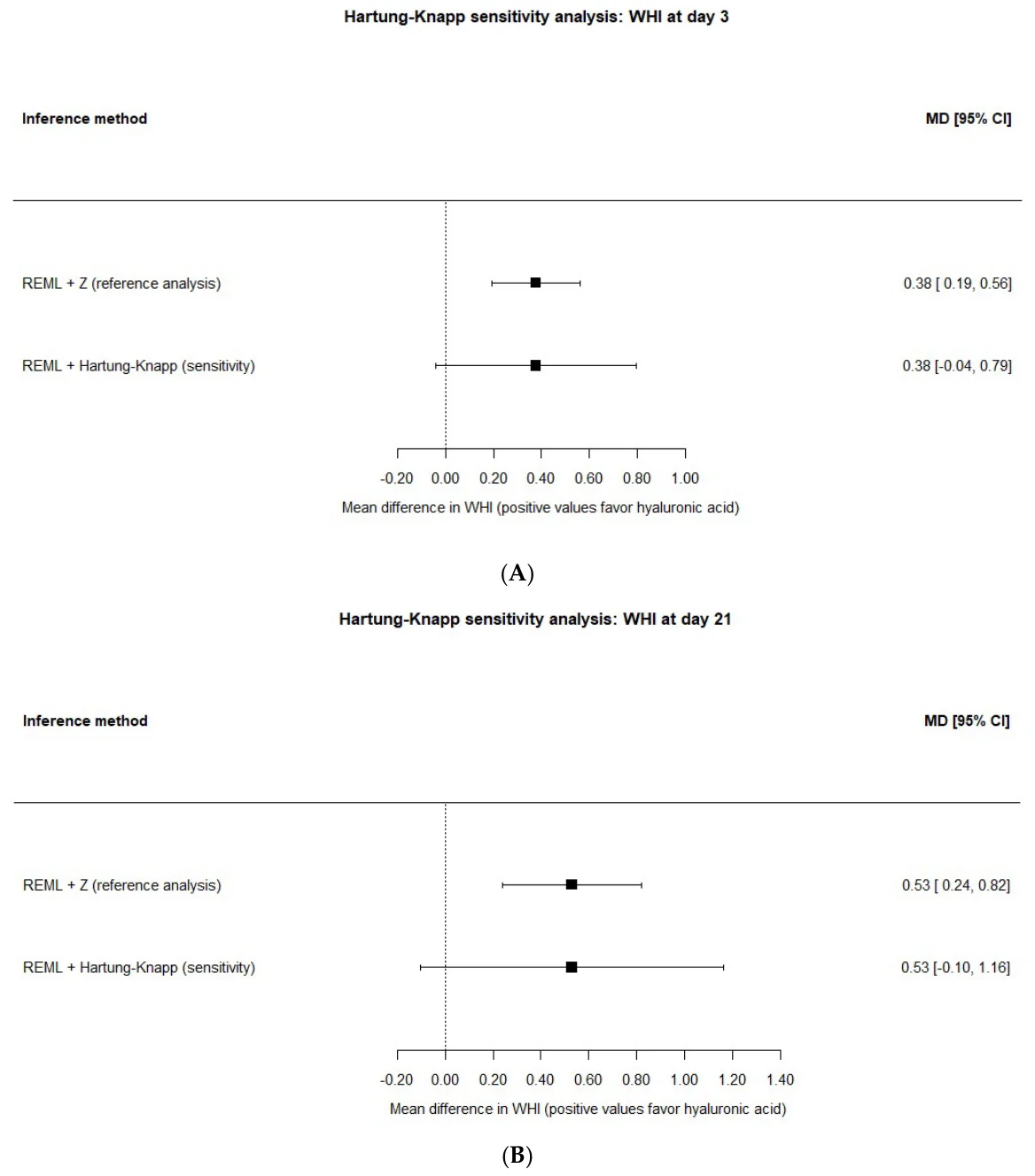
**Hartung–Knapp sensitivity analyses for the Wound Healing Index.** (**A**) Comparison of the conventional Z-based and Hartung–Knapp-adjusted estimates at postoperative day 3. (**B**) Corresponding comparison at postoperative day 21. Positive mean differences favor topical hyaluronic acid. CI, confidence interval; HA, hyaluronic acid; MD, mean difference; REML, restricted maximum likelihood; WHI, Wound Healing Index.

**Figure 9:**
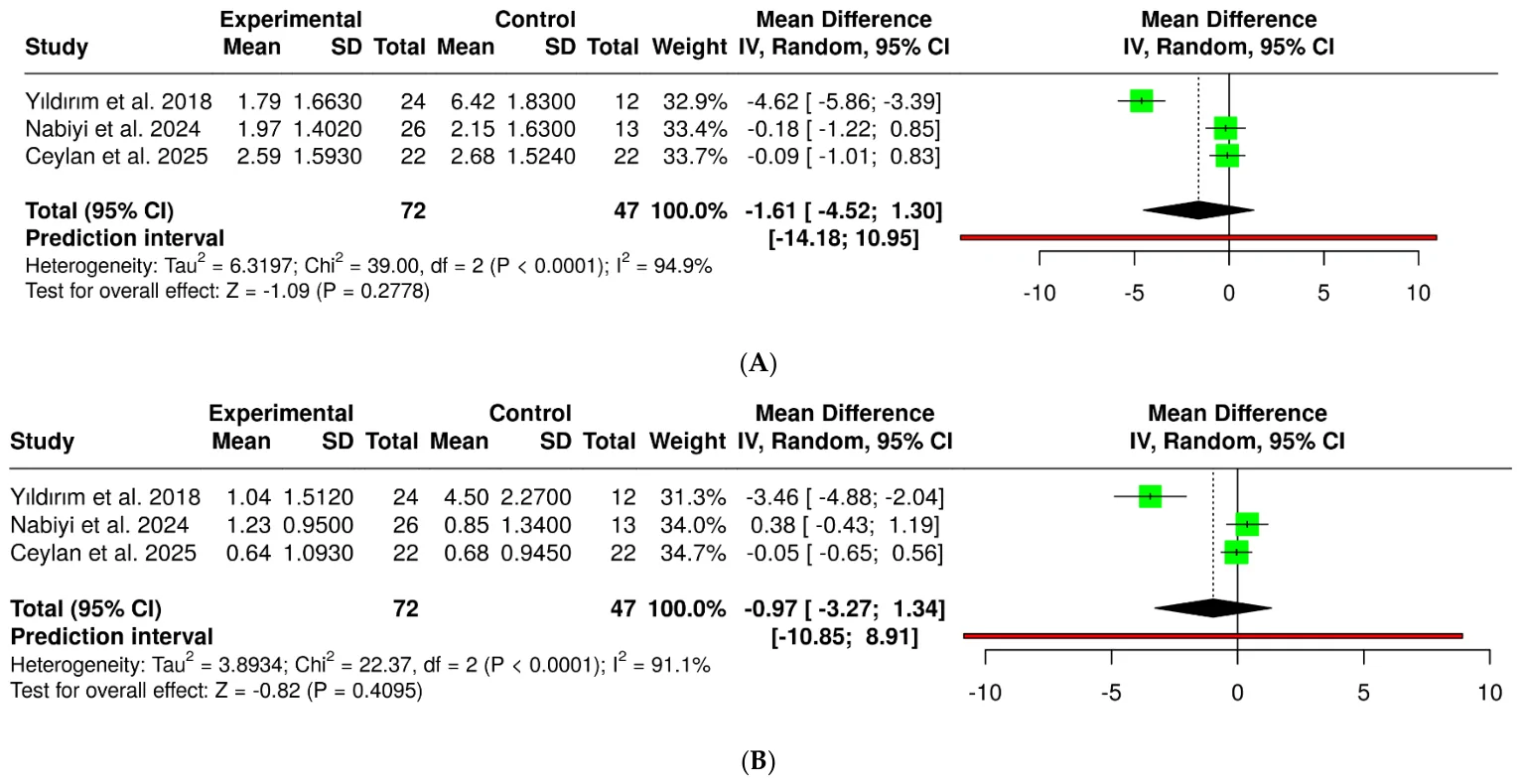
(**A**). Effect of topical hyaluronic acid on postoperative pain at day 3. (**B**). Effect of topical hyaluronic acid on postoperative pain at day 7 [10,17,22].

**Figure 10:**
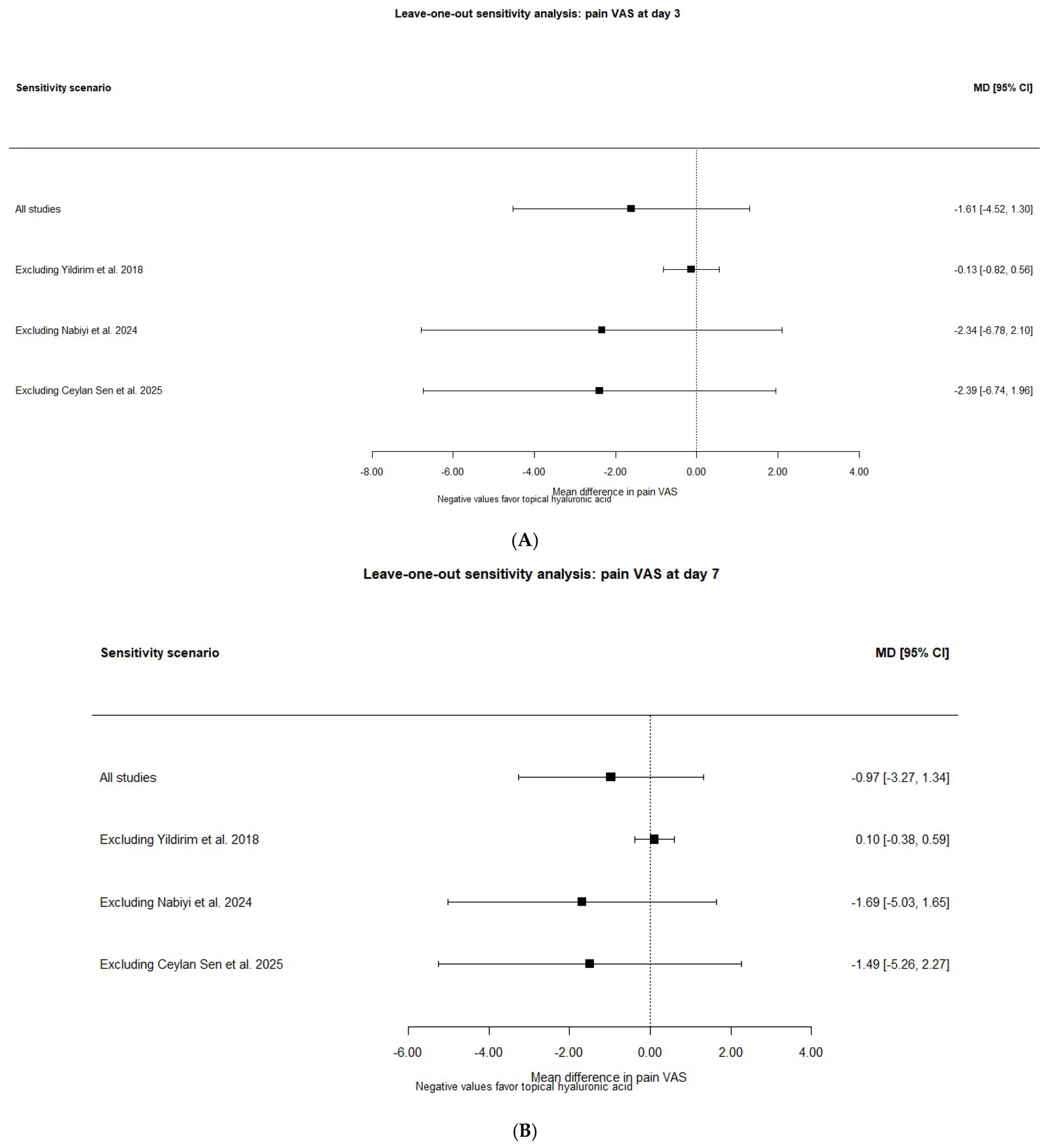
**Leave-one-out sensitivity analyses for postoperative pain.** (**A**) Postoperative day 3. (**B**) Postoperative day 7. Each analysis presents the pooled mean difference after sequential exclusion of each individual study. Negative mean differences favor topical hyaluronic acid, whereas positive values favor the control intervention. CI, confidence interval; HA, hyaluronic acid; MD, mean difference; VAS, visual analogue scale [10,17,22].

**Figure 11:**
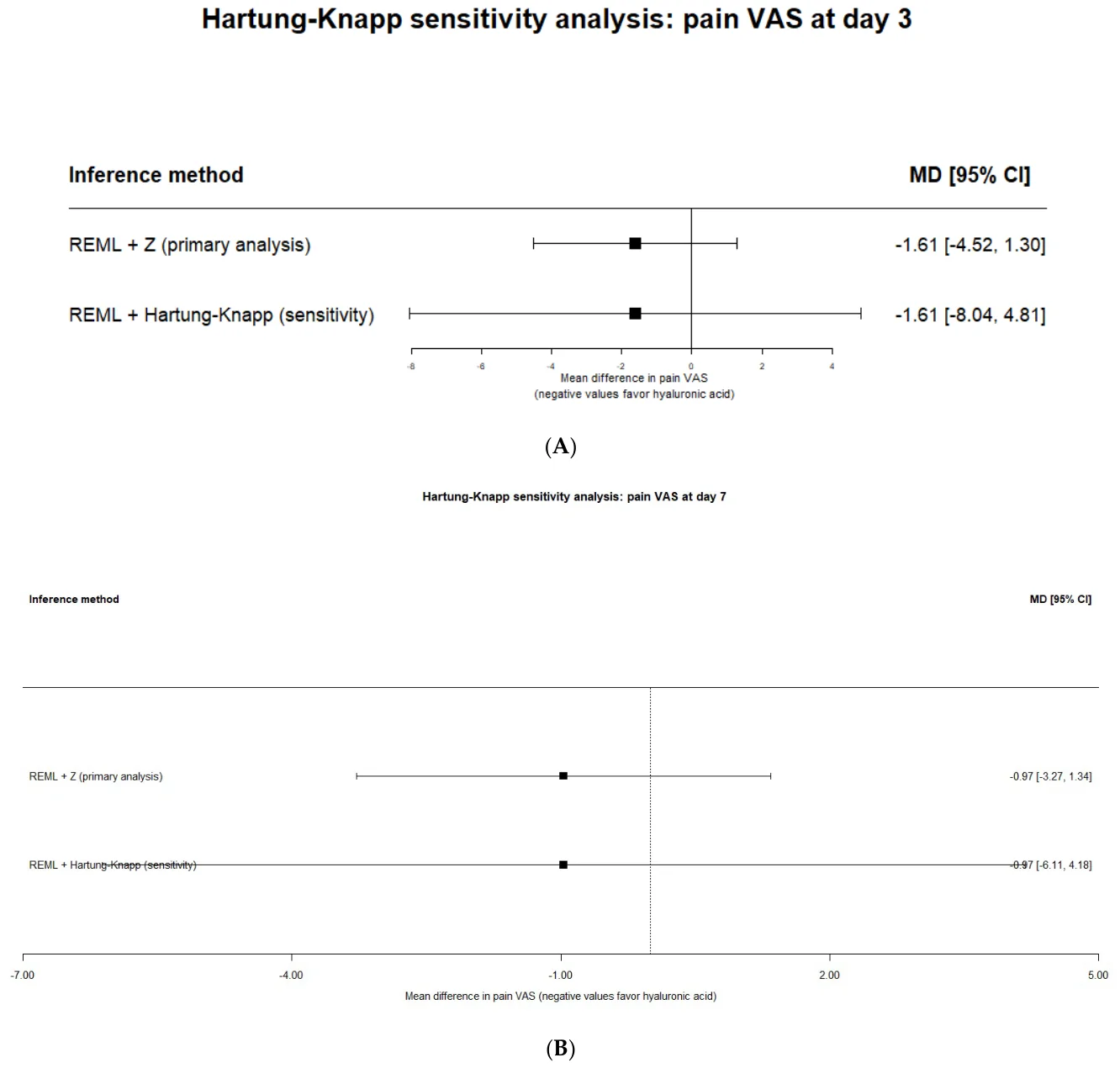
**Hartung–Knapp sensitivity analyses for postoperative pain.** (**A**) Comparison of conventional Z-based and Hartung–Knapp-adjusted estimates at postoperative day 3. (**B**) Corresponding comparison at postoperative day 7. Negative mean differences favor topical hyaluronic acid, whereas positive values favor the control intervention. CI, confidence interval; HA, hyaluronic acid; MD, mean difference; REML, restricted maximum likelihood; VAS, visual analogue scale.

**Figure 12:**
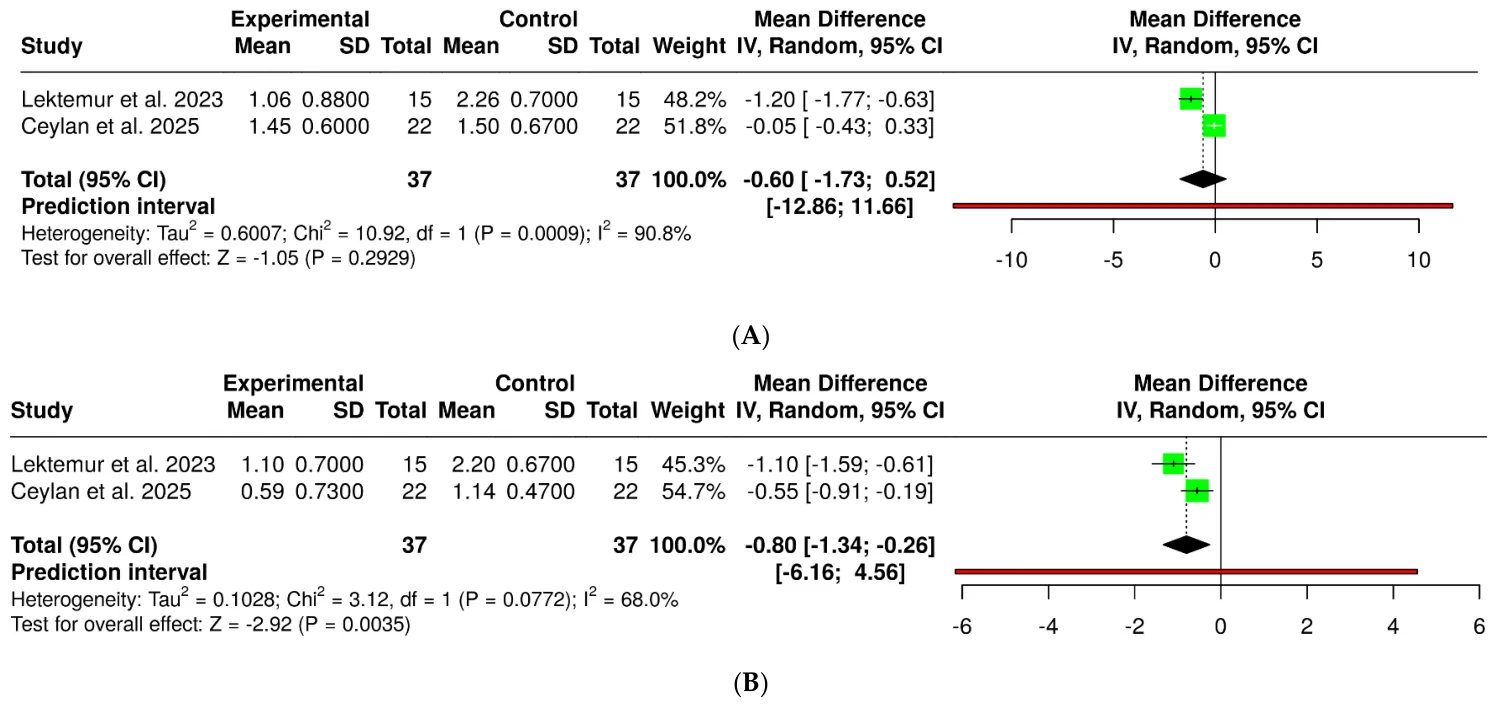
(**A**). Effect of topical hyaluronic acid on analgesic consumption at postoperative day 1. (**B**). Effect of topical hyaluronic acid on analgesic consumption at postoperative day 3 [13,17].

**Figure 13:**
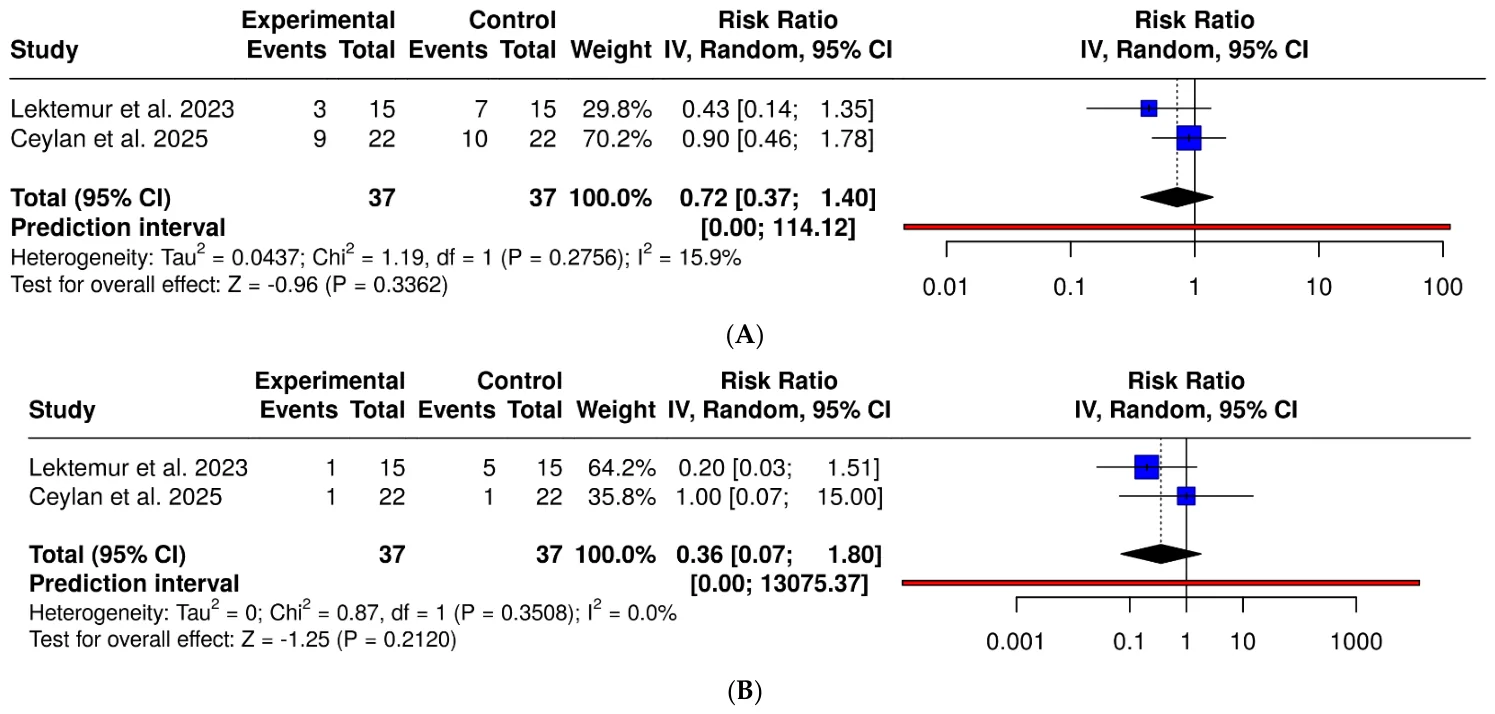
(**A**)**. Effect of topical hyaluronic acid on postoperative bleeding at day 1.** Forest plot showing risk ratios (RRs) and 95% confidence intervals calculated using a random-effects model. An RR below 1 favors topical hyaluronic acid. (**B**)**. Effect of topical hyaluronic acid on postoperative bleeding at day 3.** Forest plot showing risk ratios (RRs) and 95% confidence intervals calculated using a random-effects model. An RR below 1 favors topical hyaluronic acid [13,17].

**Figure 14:**
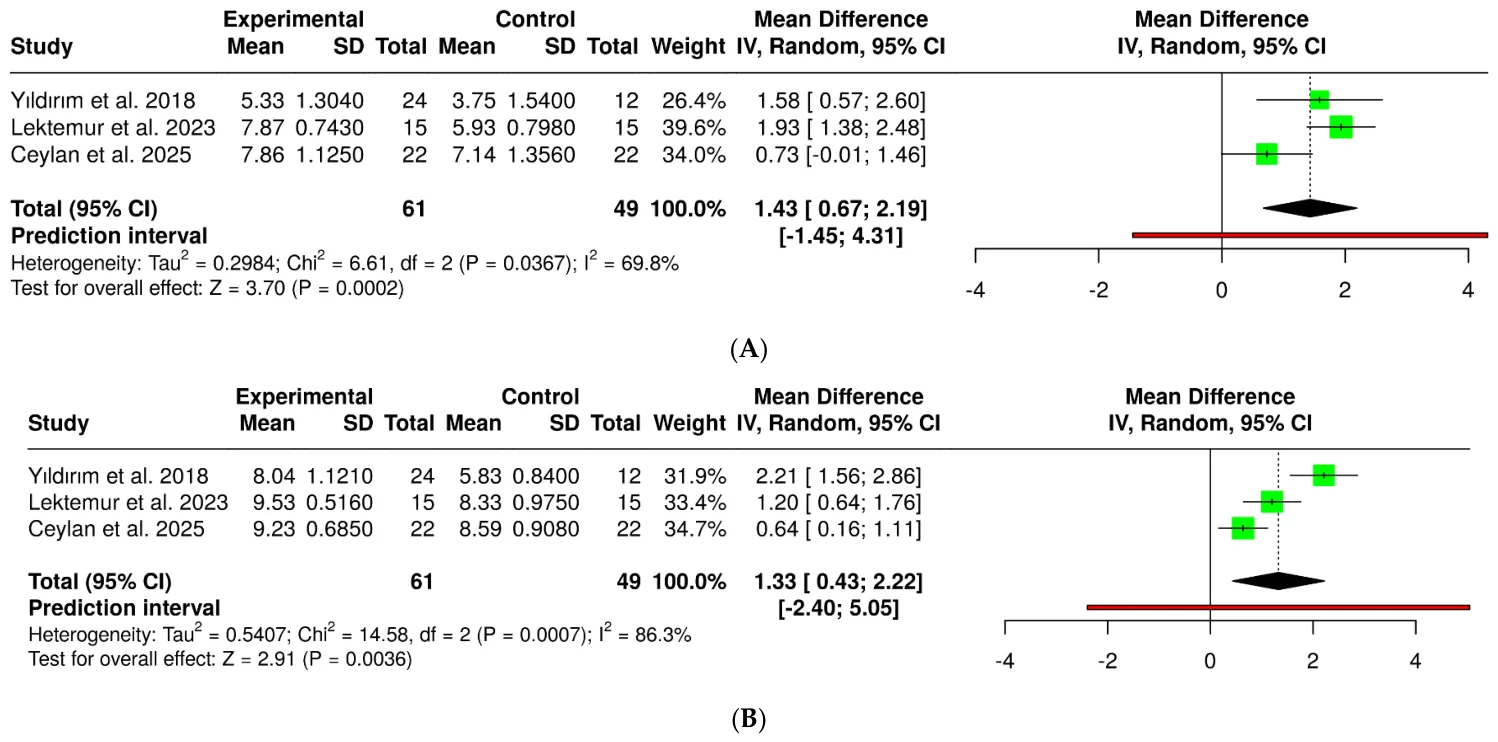
(**A**)**. Effect of topical hyaluronic acid on palatal tissue color matching at postoperative day 14.** Forest plot showing mean differences (MDs) and 95% confidence intervals calculated using a random-effects model. Color-match scores were standardized to a 0–10 scale, with positive values favoring topical hyaluronic acid. (**B**)**. Effect of topical hyaluronic acid on palatal tissue color matching at postoperative day 21.** Forest plot showing mean differences (MDs) and 95% confidence intervals calculated using a random-effects model. Color-match scores were standardized to a 0–10 scale, with positive values favoring topical hyaluronic acid [10,13,17].

**Figure 15:**
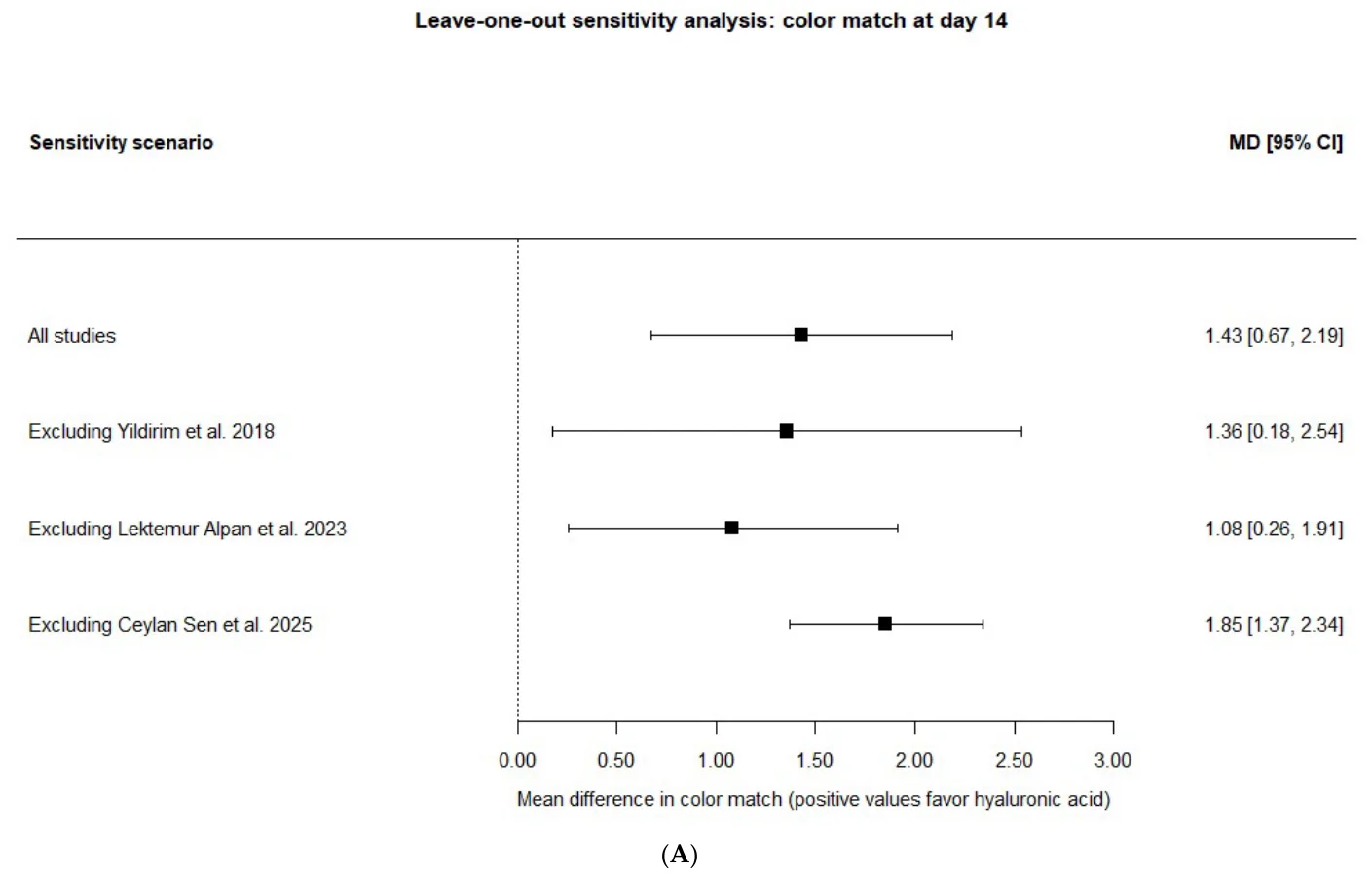

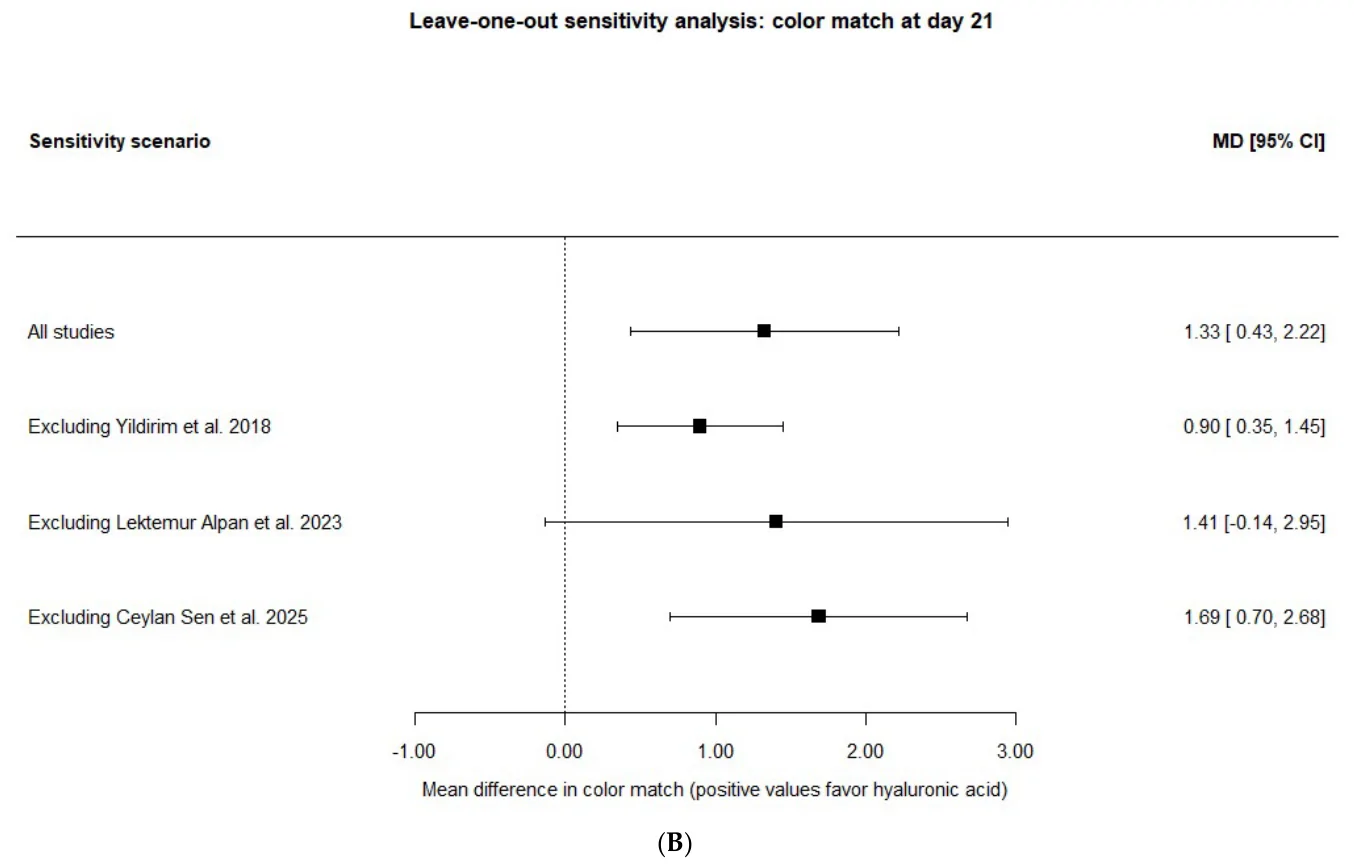
**Leave-one-out sensitivity analyses for palatal tissue color match.** (**A**) Postoperative day 14. (**B**) Postoperative day 21. Each analysis presents the pooled mean difference after sequential exclusion of each individual study. Positive mean differences favor topical hyaluronic acid, whereas negative values favor the control intervention. CI, confidence interval; HA, hyaluronic acid; MD, mean difference [10,13,17].

**Figure 16:**
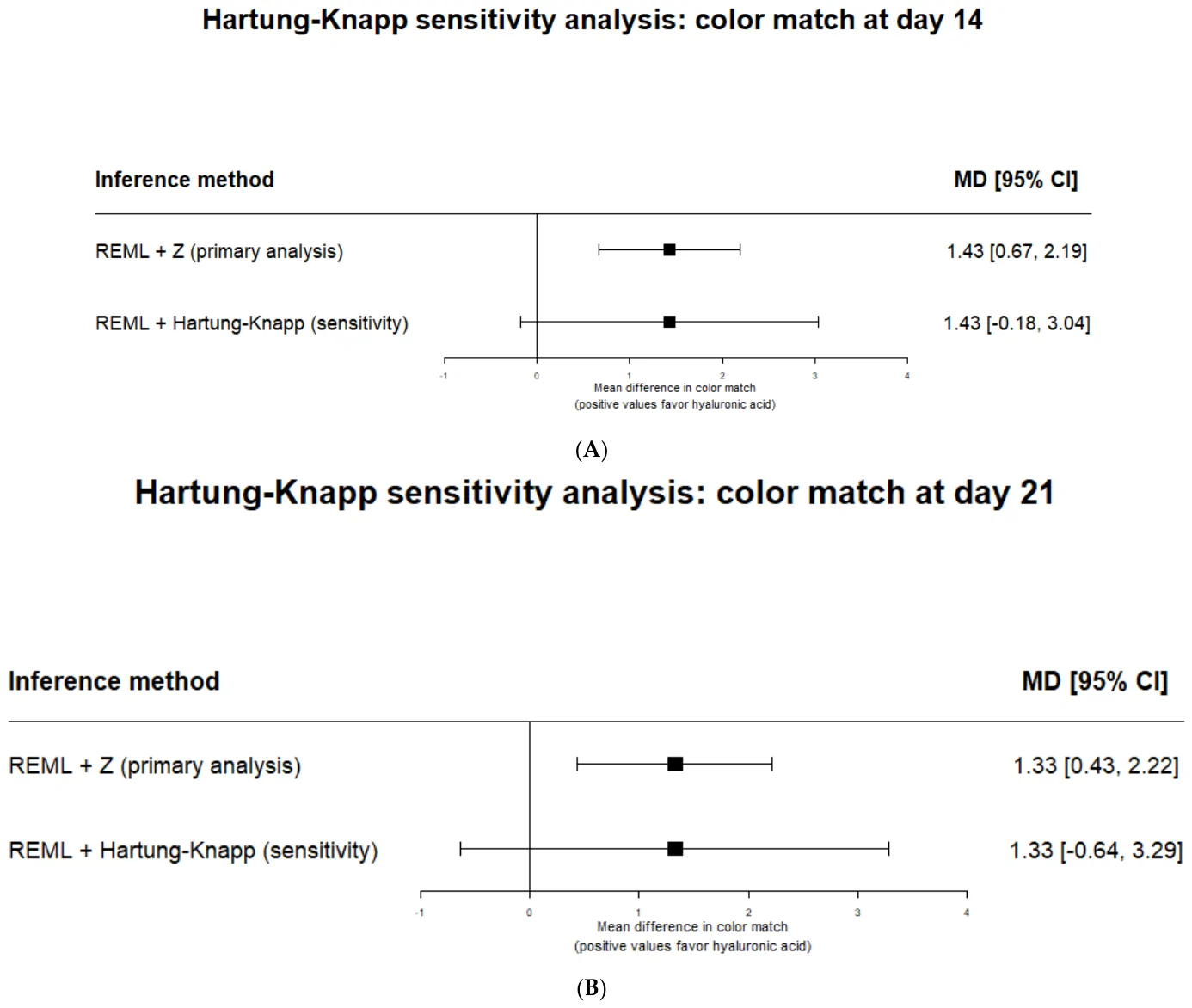
**Hartung–Knapp sensitivity analyses for palatal tissue color match.** (**A**) Comparison of conventional Z-based and Hartung–Knapp-adjusted estimates at postoperative day 14. (**B**) Corresponding comparison at postoperative day 21. Positive mean differences favor topical hyaluronic acid, whereas negative values favor the control intervention. CI, confidence interval; HA, hyaluronic acid; MD, mean difference; REML, restricted maximum likelihood.

**Figure 17:**
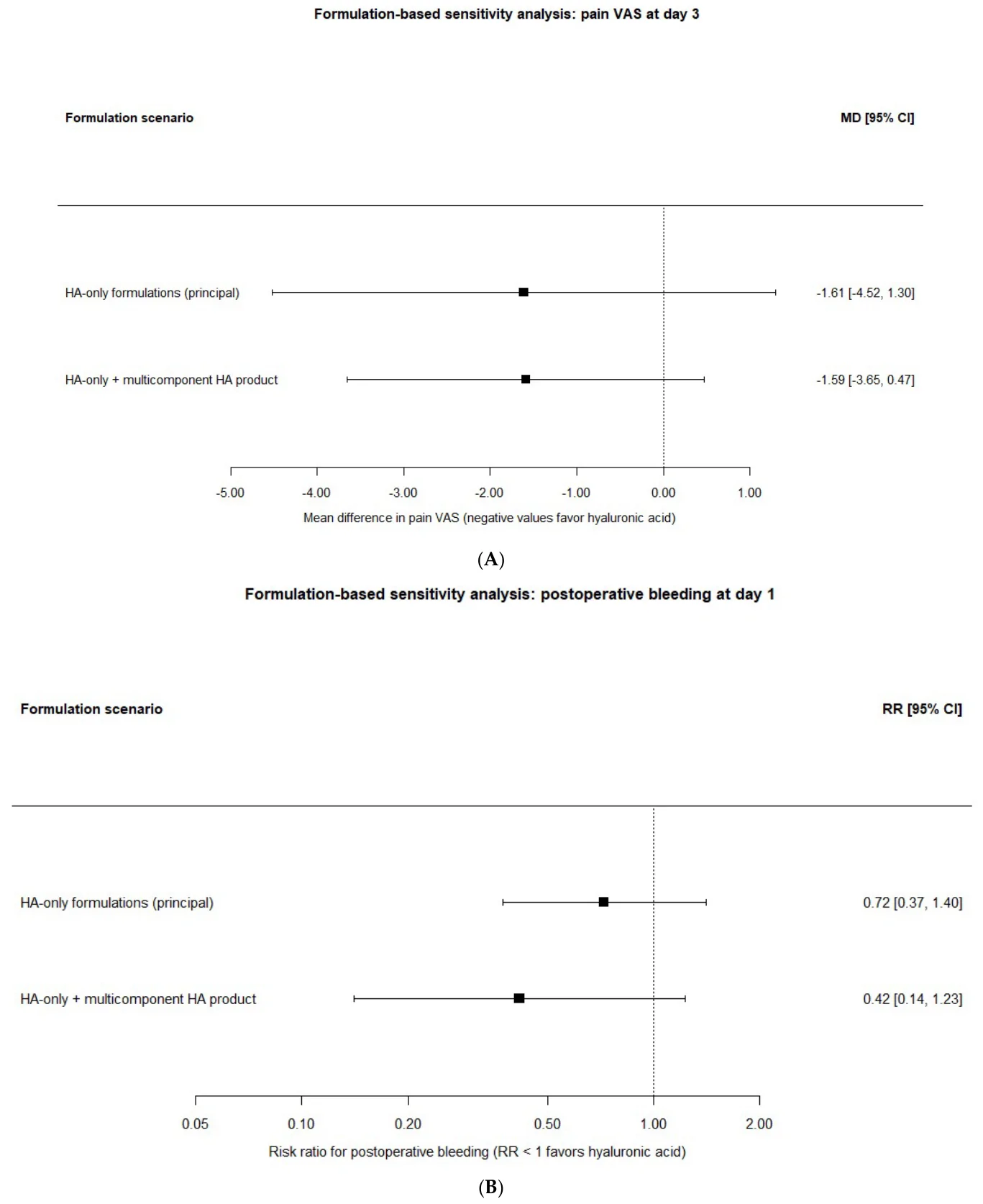

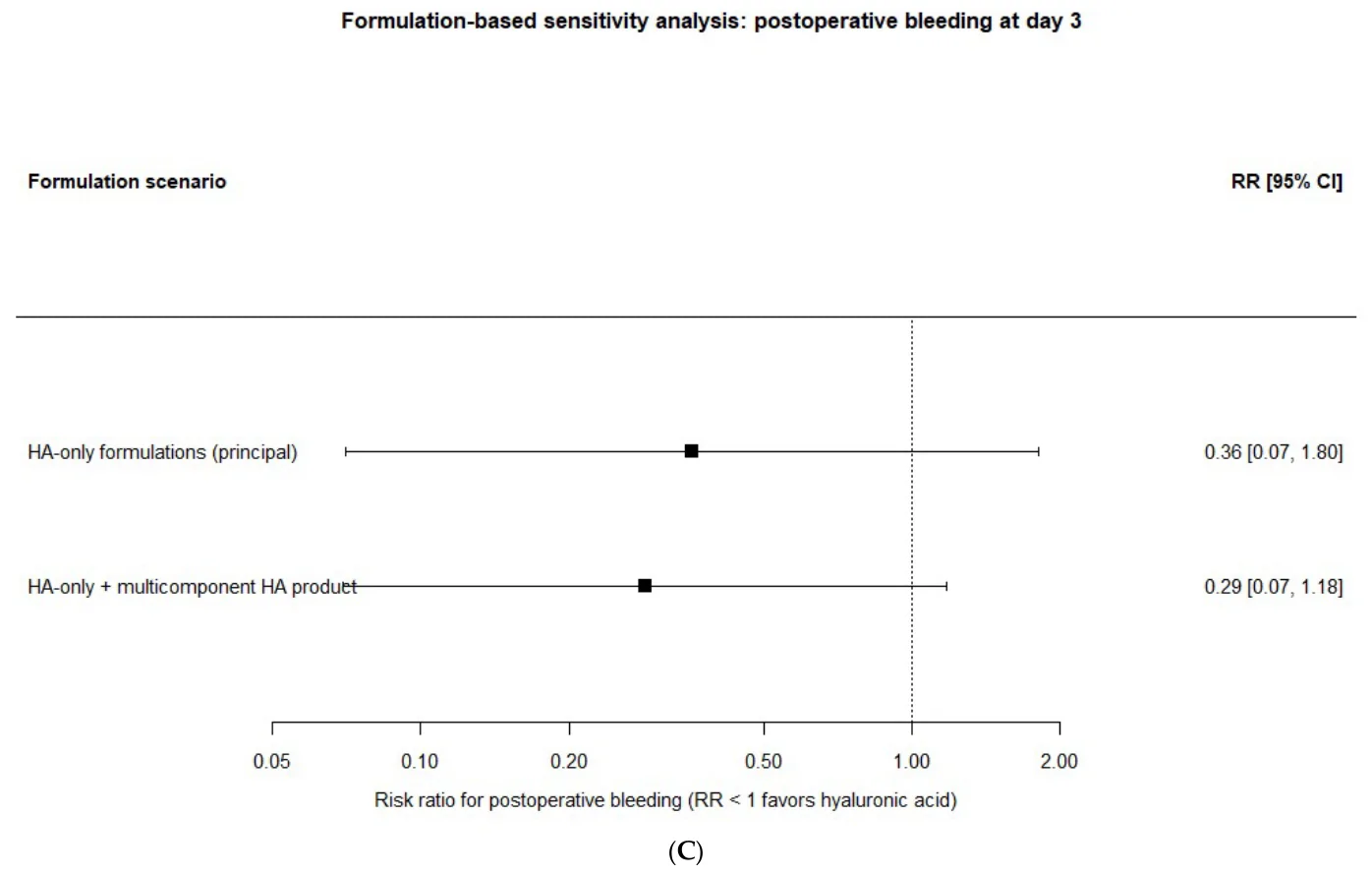
**Sensitivity analyses according to hyaluronic acid formulation.** (**A**) Postoperative pain measured using a visual analogue scale at day 3. (**B**) Postoperative bleeding at day 1. (**C**) Postoperative bleeding at day 3. The principal analyses restricted to formulations containing HA as the sole active therapeutic component were compared with broader analyses that additionally included multicomponent HA-containing products. Negative mean differences favor topical HA for postoperative pain, whereas risk ratios below 1 favor HA for postoperative bleeding. CI, confidence interval; HA, hyaluronic acid; MD, mean difference; RR, risk ratio; VAS, visual analogue scale.

**Figure 18:**
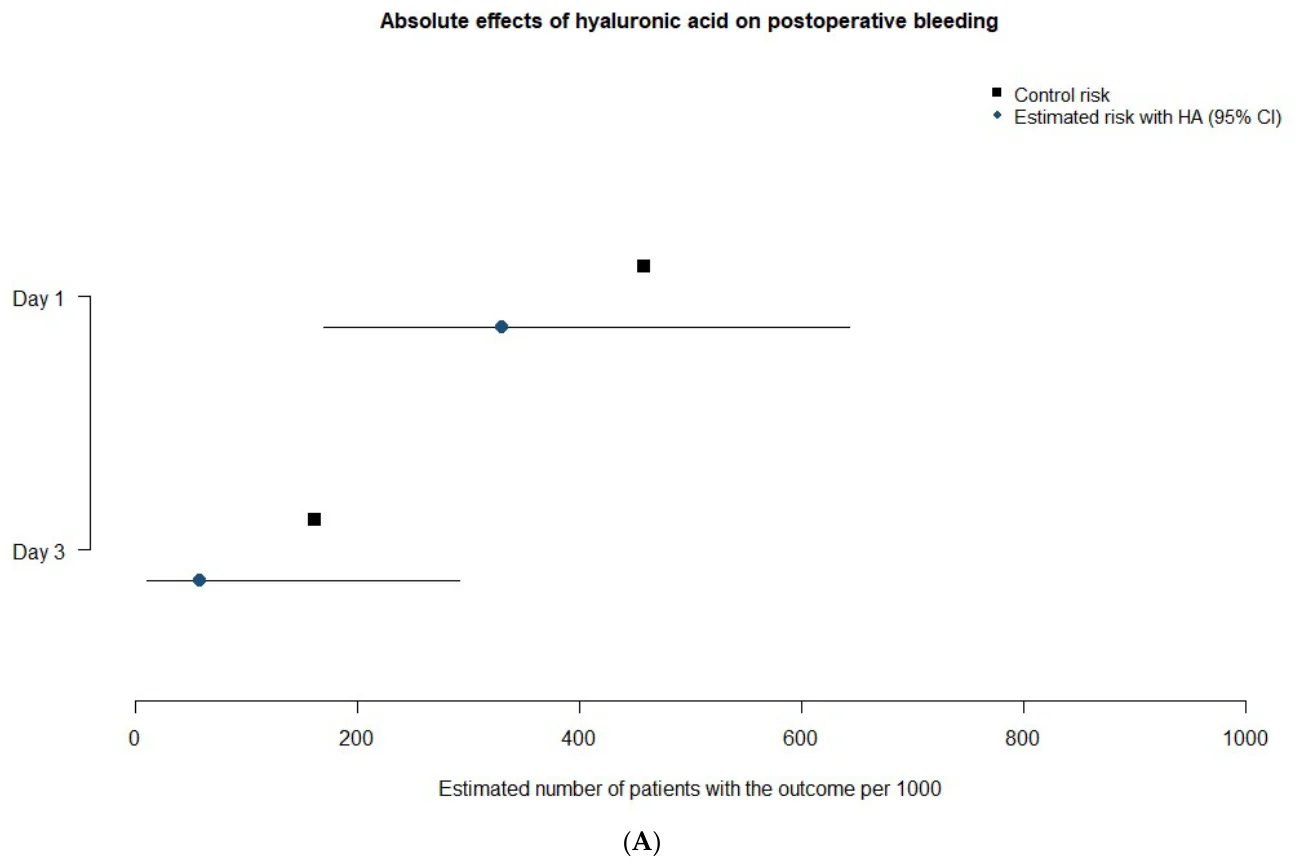

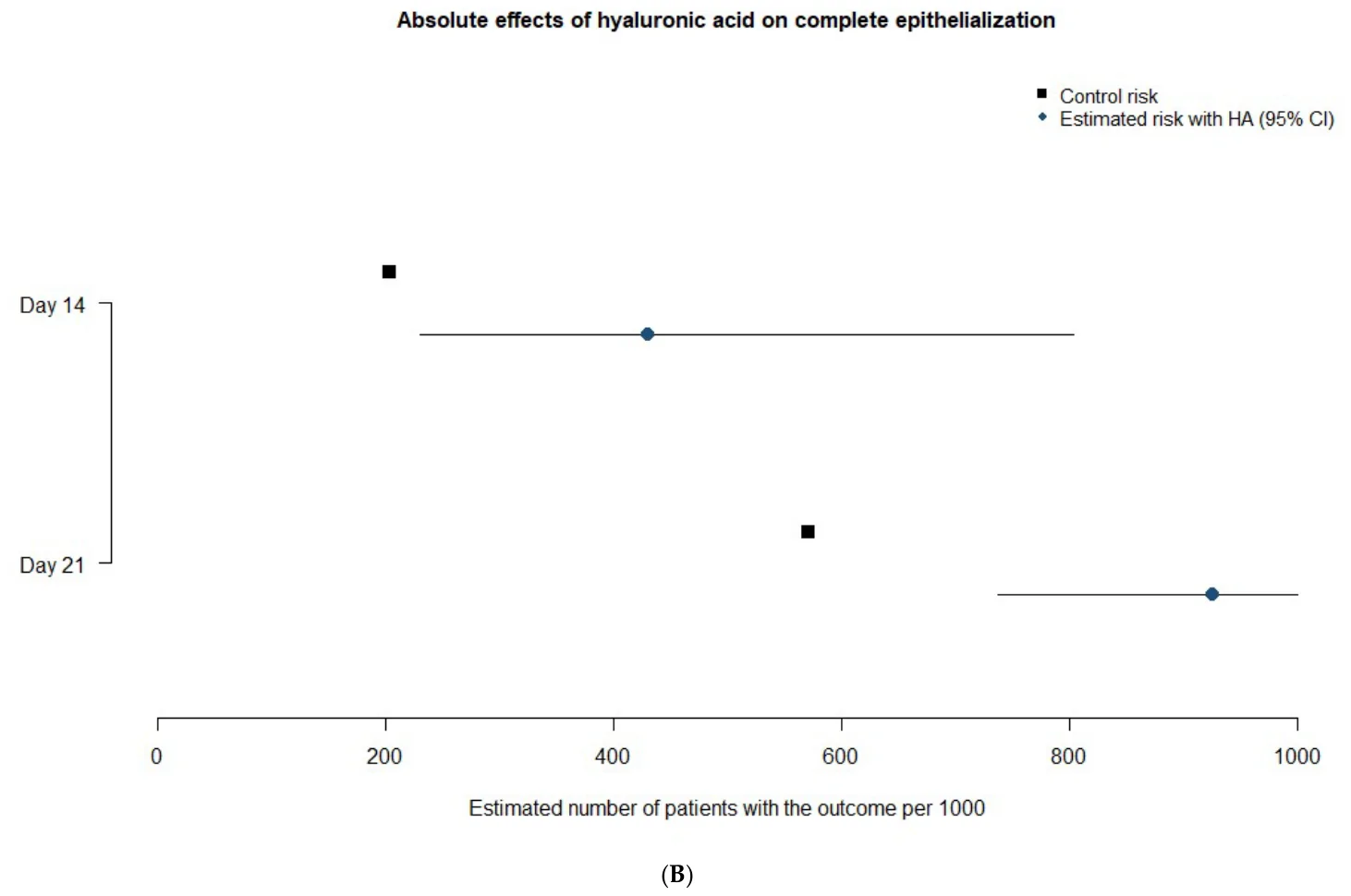
Absolute effects of topical hyaluronic acid on postoperative bleeding and complete epithelialization. (**A**) Estimated absolute effects on postoperative bleeding at days 1 and 3. (**B**) Estimated absolute effects on complete epithelialization at postoperative days 14 and 21. Absolute effects are expressed as estimated events per 1000 patients based on the observed control-group risks and pooled risk ratios. Error bars represent 95% confidence intervals. HA, hyaluronic acid; NNTB, number needed to treat for one additional beneficial outcome; RR, risk ratio.

**Table 1:** Characteristics of the studies included in the systematic review and meta-analysis.

First Author	Year	Country	Study Design	Sample Analyzed	HA Intervention	Comparator	Follow-Up	Outcomes Assessed	Main Findings/Conclusion
Yıldırım et al. [10]	2018	Türkiye	Examiner-masked, parallel RCT; 3 arms	36: HA 0.2% *n* = 12; HA 0.8% *n* = 12; control *n* = 12	Gengigel 0.2% or 0.8% applied to the palatal donor site, covered with periodontal dressing and reapplied on day 3	Periodontal dressing alone	Days 3, 7, 14, 21 and 42	Pain and burning VAS, complete epithelialization, color match, adverse events	Both HA concentrations reduced early pain and burning. Complete epithelialization was achieved in all HA-treated patients by day 21, whereas the control group reached complete epithelialization by day 42.
Hassan et al. [11]	2021	Egypt	Parallel RCT; 3 arms	30 analyzed: MEBO *n* = 10; HA *n* = 10; control *n* = 10. Thirty-nine randomized	Gengigel 0.2% applied to the palatal wound, covered with periodontal pack and reapplied on day 3	Periodontal pack alone; additional active MEBO arm	Pain: days 1–7; wound area: baseline and days 3, 7, 14 and 21; color match to day 42	Pain VAS, analgesic consumption, palatal wound area and color match	HA reduced postoperative pain and total analgesic consumption compared with control. No significant intergroup differences were found for palatal wound area. MEBO produced greater pain reduction than HA.
Khalil et al. [12]	2022	Jordan	Parallel RCT; 2 arms	24 randomized; 21 complete according to text: HA *n* = 11, control *n* = 10; CONSORT figures are inconsistent	HYADENT BG applied once to the donor and recipient sites and injected into the collagen sponge placed over the palatal wound	Saline applied to both sites; saline-soaked collagen sponge. Both groups received periodontal dressing	Pain for 14 days; recipient graft area at baseline, 1, 3 and 6 months	Donor- and recipient-site pain; recipient graft area; color match and satisfaction	HA reduced donor-site pain during the first 7 days. No significant effect was found on recipient-site graft dimensions, color match or satisfaction. The study does not provide objective palatal epithelialization data.
Lektemur Alpan et al. [13]	2023	Türkiye	Single-blind, parallel RCT; 4 arms	60: HA *n* = 15; control *n* = 15; HOCl *n* = 15; flurbiprofen *n* = 15	High-molecular-weight sodium hyaluronate gel, 600 mg/100 g (0.6%), applied twice daily for 14 days	No topical agent; additional HOCl and flurbiprofen arms	Days 1, 3, 7, 14, 21 and 28	WHI, complete epithelialization, H_2_O_2_ bubbling, pain, burning, chewing discomfort, color match, analgesic consumption and delayed bleeding	HA improved WHI and accelerated complete epithelialization. At day 21, epithelialization was complete in 15/15 HA patients versus 10/15 controls. HA also reduced analgesic consumption and delayed bleeding.
Baroudi et al. [15]	2024	Syria	Parallel RCT; 2 arms	18: PVP-SH *n* = 9; control *n* = 9	Gelclair PVP-sodium hyaluronate gel, 15 mL inside a plastic stent, three times daily for 1 week	Coe-Pak periodontal dressing	Weekly for 1 month; pain and analgesic use during first 7 days	Pain VAS, analgesic consumption, complete epithelialization, Landry healing scale and willingness to undergo retreatment	The PVP-SH group showed less pain, lower analgesic use and faster healing than Coe-Pak. Interpretation is limited because Gelclair is multicomponent and the groups received different mechanical coverings.
Nabiyi et al. [22]	2024	Iran	Triple-blind, parallel RCT; 3 arms	39: HA 0.2% *n* = 13; HA 0.8% *n* = 13; vehicle control *n* = 13	Mucoadhesives containing HA 0.2% or 0.8%; applied to the palatal donor site and protected with periodontal dressing	Identical mucoadhesive vehicle without HA plus periodontal dressing	Days 3, 7, 14, 21 and 42	Pain VAS, thermal sensitivity, Landry WHI and modified Manchester Scar Proforma	HA-containing mucoadhesives improved wound-healing characteristics, particularly with 0.8% HA at day 21. No significant differences were observed in postoperative pain between groups.
Bulut et al. [16]	2025	Türkiye	Prospective, parallel RCT; 2 arms	32: Gelclair *n* = 16; control *n* = 16	Gelclair PVP-sodium hyaluronate gel twice daily for 14 days plus 0.12% chlorhexidine	0.12% chlorhexidine alone	Days 1, 3, 7, 14 and 28	H_2_O_2_ bubbling, WHI, pain, burning, chewing discomfort and delayed bleeding	Gelclair improved WHI and H_2_O_2_ epithelialization findings and reduced postoperative discomfort and early bleeding. Because Gelclair contains PVP, sodium hyaluronate and glycyrrhetinic acid, the effect cannot be attributed exclusively to HA.
Ceylan et al. [17]	2025	Türkiye	Examiner-masked, parallel RCT; 3 arms	67 analyzed: HA *n* = 22; I-PRF *n* = 23; control *n* = 22	Gelatin sponge saturated once with 0.8% PVP-sodium hyaluronate gel and sutured to the palatal wound; acrylic stent for 3 days	Sterile gelatin sponge; additional I-PRF-impregnated sponge arm	Days 3, 7, 14, 21 and 28; tissue thickness and related outcomes to 3 months	WHI, complete epithelialization/H_2_O_2_ bubbling, pain, burning, analgesic use, bleeding, color match, tissue consistency and OHRQoL	HA and I-PRF improved healing and reduced morbidity compared with sponge alone. HA generally produced the most favorable clinical wound-healing outcomes.

## Data Availability

The raw data supporting the conclusions of this article will be made available by the authors, without undue reservation.

## Supplementary Materials

The following supporting information can be downloaded at: https://www.mdpi.com/article/10.3390/jpm16100492/s1: Table S1: PRISMA 2020 checklist; Table S2: Complete electronic search strategies for all databases and information sources, including final search dates and numbers of records retrieved.
