# Supplementary material for: Topical Hyaluronic Acid for Palatal Donor-Site Healing After Autogenous Gingival Graft Harvesting: A Systematic Review and Time-Specific Meta-Analysis with Clinical Implications for Postoperative Care
Source: J Pers Med. 2026 Sep 23;16(10):492. doi: 10.3390/jpm16100492 (PMC13705756; doi:10.3390/jpm16100492)
Supplement: Supplementary file 1 [file jpm-16-00492-s001.zip › Table S2.pdf]

Table S2. Complete Electronic Search Strategies for All Databases and Information Sources, Including Final Search Dates and Numbers of Records Retrieved

Database / Information Source	Search Strategy
**MEDLINE via PubMed**	("Hyaluronic Acid"[MeSH Terms] OR "hyaluronic acid"[Title/Abstract] OR hyaluronan[Title/Abstract] OR "sodium hyaluronate"[Title/Abstract]) AND ("Palate"[MeSH Terms] OR palatal[Title/Abstract] OR palate[Title/Abstract]) AND ("Gingival Transplantation"[MeSH Terms] OR "Transplant Donor Site"[MeSH Terms] OR "free gingival graft"[Title/Abstract] OR "connective tissue graft"[Title/Abstract] OR "soft tissue graft"[Title/Abstract] OR "donor site"[Title/Abstract] OR harvesting[Title/Abstract] OR wound[Title/Abstract])
**Embase**	('hyaluronic acid'/exp OR 'hyaluronic acid':ti,ab OR hyaluronan:ti,ab OR 'sodium hyaluronate':ti,ab) AND ('palate'/exp OR palatal:ti,ab OR palate:ti,ab) AND ('gingival graft':ti,ab OR 'free gingival graft':ti,ab OR 'connective tissue graft':ti,ab OR 'soft tissue graft':ti,ab OR 'donor site':ti,ab OR harvesting:ti,ab OR wound:ti,ab)
**Web of Science Core Collection**	TS=(("hyaluronic acid" OR hyaluronan OR "sodium hyaluronate") AND (palate OR palatal) AND ("free gingival graft" OR "connective tissue graft" OR "soft tissue graft" OR "donor site" OR harvesting OR wound))
**Cochrane Oral Health Group Trials Register**	("hyaluronic acid" OR hyaluronan OR "sodium hyaluronate") AND (palate OR palatal) AND ("free gingival graft" OR "connective tissue graft" OR "soft tissue graft" OR "donor site" OR harvesting OR wound)
**Latindex**	("hyaluronic acid" OR "ácido hialurónico" OR hyaluronan OR "sodium hyaluronate") AND (palate OR palatal OR paladar) AND ("gingival graft" OR "injerto gingival" OR "donor site" OR "sitio donante" OR wound OR herida)
**SciELO**	("hyaluronic acid" OR "ácido hialurónico" OR hyaluronan OR "sodium hyaluronate") AND (palate OR palatal OR paladar) AND ("free gingival graft" OR "injerto gingival libre" OR "connective tissue graft" OR "injerto de tejido conectivo" OR "donor site" OR "sitio donante" OR wound OR herida)
